# Tailored tetravalent antibodies potently and specifically activate Wnt/Frizzled pathways in cells, organoids and mice

**DOI:** 10.7554/eLife.46134

**Published:** 2019-08-27

**Authors:** Yuyong Tao, Monika Mis, Levi Blazer, Mart Ustav, Zachary Steinhart, Rony Chidiac, Elli Kubarakos, Siobhan O'Brien, Xiaomei Wang, Nick Jarvik, Nish Patel, Jarrett Adams, Jason Moffat, Stephane Angers, Sachdev S Sidhu

**Affiliations:** 1 Donnelly CentreUniversity of TorontoTorontoCanada; 2 Department of Pharmaceutical SciencesLeslie Dan Faculty of Pharmacy, University of TorontoTorontoCanada; 3 Department of BiochemistryUniversity of TorontoTorontoCanada; 4 Department of Molecular GeneticsUniversity of TorontoTorontoCanada; 5 Canadian Institute for Advanced ResearchTorontoCanada; California Institute of TechnologyUnited States; California Institute of TechnologyUnited States

**Keywords:** antibody, Wnt, frizzled, regenerative medicine, organoid, pluripotent stem cell, Mouse

## Abstract

Secreted Wnt proteins regulate development and adult tissue homeostasis by binding and activating cell-surface Frizzled receptors and co-receptors including LRP5/6. The hydrophobicity of Wnt proteins has complicated their purification and limited their use in basic research and as therapeutics. We describe modular tetravalent antibodies that can recruit Frizzled and LRP5/6 in a manner that phenocopies the activities of Wnts both in vitro and in vivo. The modular nature of these synthetic Frizzled and LRP5/6 Agonists, called FLAgs, enables tailored engineering of specificity for one, two or multiple members of the Frizzled family. We show that FLAgs underlie differentiation of pluripotent stem cells, sustain organoid growth, and activate stem cells in vivo. Activation of Wnt signaling circuits with tailored FLAgs will enable precise delineation of functional outcomes directed by distinct receptor combinations and could provide a new class of therapeutics to unlock the promise of regenerative medicine.

## Introduction

Wnt proteins are secreted glycoproteins that regulate stem and progenitor cells, and thus play fundamental roles in development and adult tissue homeostasis (Steinhart and Angers, 2018; Wiese et al., 2018). In humans, 19 Wnt proteins interact with a receptor complex composed of one of ten Frizzled (FZD) receptors and one of several co-receptors that guide the selective engagement of different intracellular signaling branches (Angers and Moon, 2009; Wodarz and Nusse, 1998). In the best characterized pathway, Wnt interacts with a FZD and a single transmembrane LRP5 or LRP6 co-receptor to stabilize intracellular βcatenin and regulate genes involved in tissue stem cell proliferation and differentiation. Wnt binds to the cysteine rich domain (CRD) of FZD (Janda et al., 2012) and to either the first two or last two propeller domains of LRP5/6 (Bourhis et al., 2010), and these interactions are thought to allosterically modulate the FZD seven-transmembrane domain to enable recruitment of Disheveled and allow phosphorylation of the LRP5/6 intracellular domain (Davidson et al., 2005; Zeng et al., 2005).

Wnts require lipidation for function (Janda et al., 2012; Kadowaki et al., 1996) and their hydrophobic nature complicates biochemical manipulation; consequently, only a few Wnts have been purified (Willert et al., 2003). Furthermore, Wnts are inherently cross-reactive for multiple receptors, especially when overexpressed or applied at high dose (Dijksterhuis et al., 2015; He et al., 1997; Holmen et al., 2002). As a result, it has been impossible to activate Frizzled receptor complexes selectively to determine the specific functions of each in different contexts or to evaluate their therapeutic potential for degenerative conditions. Thus, water-soluble synthetic Wnt analogs with tailored specificities would have great significance for both basic research and regenerative medicine.

Recently, bivalent Wnt surrogates have been developed by fusing an LRP5/6-binding fragment from the natural protein DKK1 to an engineered ligand for FZD CRDs (Janda et al., 2017). These Wnt surrogates activated βcatenin signaling in vitro and in vivo when delivered using adenovirus. Herein, we report modular and engineerable FZD/LRP agonists (FLAgs): tetravalent synthetic antibodies (Abs) that enable selective and robust activation of any FZD receptor in vitro and in vivo. Leveraging a panel of hundreds of synthetic Abs targeting FZDs and their co-receptors, we built FLAgs for selective and rational activation of one, two or multiple FZD receptors. FLAgs are composed of human antibody domains, and consequently, they are highly stable, amenable to large-scale production and facile purification, have predictable pharmacokinetics, and are likely to exhibit low immunogenicity. We thus anticipate that tailored FLAgs will improve directed differentiation and cell therapy endeavors, sustain tissue organoid growth, and enable therapeutic mobilization of endogenous stem cells in vivo to promote tissue repair after injury and restore function following tissue degeneration.

## Results

### Synthetic antibodies targeting FZD and LRP6

**Figure 1.**
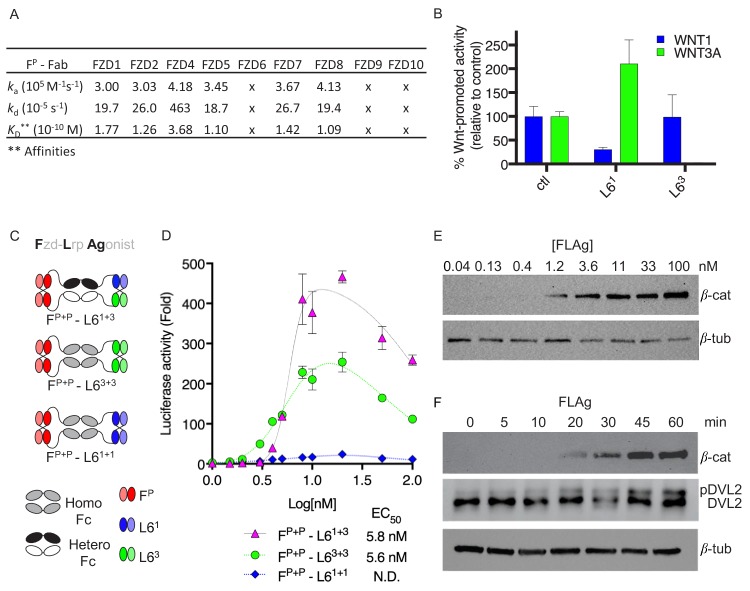
Design and validation of FLAgs as activators of the Wnt-βcatenin pathway. (**A**) Surface plasmon resonance (SPR) binding kinetics of F^P^ Fab. Kinetics were derived from curves for soluble F^P^ Fab interacting with immobilized FZD CRD, and ‘x’ indicates no detectable binding. (**B**) Anti-LRP6 Ab inhibitory activity. Inhibition of WNT1 or WNT3A signaling by indicated LRP6 Abs in the diabody-Fc format was assessed using stimulation with purified WNT3A or upon *WNT1* cDNA transfection. (**C**) Molecular architecture of tetravalent FLAgs. (**D**) Activation of βcatenin signaling by FLAgs. Dose response curves are shown for the activation of a LEF/TCF reporter gene (*y-axis*) in HEK293T cells by serial dilutions of pan-specific FLAg proteins (F^P+P^-L6^1+1^, F^P+P^-L6^3+3^ and F^P+P^-L6^1+3^) (*x-axis*). Error bars indicate SEM, n = 3. (**E**) Levels of βcatenin protein in RKO cells after 30 min treatment with the indicated concentrations of pan-FLAg (F^P+P^-L6^1+3^). Representative blot of three replicates. (**F**) Time course of βcatenin and phosphorylated Disheveled-2 (p-DVL2) protein levels in RKO cells treated with 10 nM pan-FLAg (F^P+P^-L6^1+3^). Representative blot of three replicates. 10.7554/eLife.46134.004Figure 1—source data 1.Source data for Figure 1B. 10.7554/eLife.46134.005Figure 1—source data 2.Source data for Figure 1B.

We previously applied phage display to derive synthetic Abs using recombinant FZD CRDs as antigens (Pavlovic et al., 2018; Steinhart et al., 2017). Systematic characterization revealed a continuum of specificity profiles with some Abs displaying broad specificities, exemplified by a pan-FZD Ab (F^P^) that recognized FZD1/2/4/5/7/8 (Figure 1A and Figure 1—figure supplement 1A), others displaying more restricted specificities, and some being monospecific.

**Figure 1—figure supplement 1.**
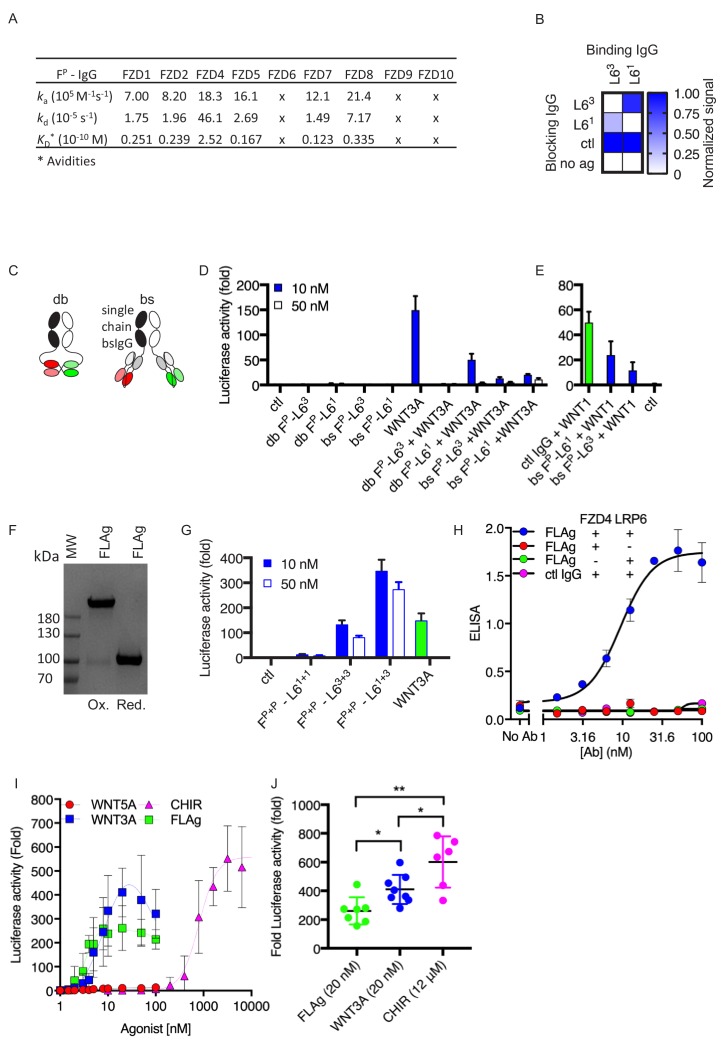
Development of Synthetic Modular Tetravalent FZD. (**A**) SPR binding kinetics of F^P^ IgG. 'x’ indicates no detectable binding. (**B**) Epitope binning for L6^1^ and L6^3^ IgG by competitive blocking measured using BLI. The normalized signal is shown for the binding of L6^1^ or L6^3^ (*x-axis*) to immobilized LRP6-Fc pre-blocked with the indicated Ab (*y-axis*). (**C**) Schematic representation of bispecific diabody-Fc (db) and single-chain bispecific IgG (bs) modalities recognizing FZD and LRP6 receptors. (**D–E**) Effects of bispecific IgGs and diabody-Fcs on Wnt-βcatenin signaling, as measured using HEK29T cells stably expressing a LEF/TCF reporter and with or without *WNT1* transient transfection or treatment with WNT3A (0.5 μg/ml). (**F**) Coomassie-stained SDS-PAGE analysis of purified FLAg F^P+P^-L6^1+3^ under oxidizing (Ox) or reducing (red) conditions. (**G**) βcatenin signaling stimulated by FLAg or WNT3A conditioned media, measured using LEF/TCF reporter assay in HEK293T cells. (**H**) Co-binding of FLAg F^P+P^-L6^1+3^ to absorbed LRP6-Fc (LRP6) and soluble concentrations of FZD4-His (FZD4) by ELISA with anti-His-HRP detection. (**I**) Dose response analysis of βcatenin signaling activity stimulated with mouse WNT3A (R and D systems 1324-WN/CF), human/mouse WNT5A (R and D systems, 645-WN/CF) purified proteins, FLAg F^P+P^-L6^1+3^ or CHIR99021. HEK293T cells stably expressing a LEF/TCF reporter were stimulated with the indicated doses for 16 hr. (**J**) The fold change of luciferase activity at maximal stimulation of Wnt reporter cells treated with 20 nM FLAg F^P+P^-L6^1+3^ , 20 nM purified WNT3A, or 12 μM CHIR99021.

We also developed two Abs that targeted the extracellular domain (ECD) of human LRP6 and bound to non-overlapping epitopes (Figure 1—figure supplement 1B). The LRP6 ECD contains four β-propeller motifs (Bao et al., 2012), and the N-terminal and C-terminal pairs interact with distinct classes of Wnt proteins represented by WNT1 or WNT3A (Bourhis et al., 2010; Gong et al., 2010), respectively. Signaling assays revealed that Abs L6^1^ and L6^3^ selectively inhibited βcatenin activation by WNT1 or WNT3A, respectively (Figure 1B), suggesting that each Ab blocks one of the two distinct Wnt binding sites on LRP6.

### Rational design of tetravalent agonists for FZD and LRP6

We first asked whether bivalent Abs could assemble active heterodimers of FZD and LRP6 by constructing bispecific modalities using the pan-FZD Ab F^P^ and anti-LRP6 Abs L6^1^ and L6^3^ (Figure 1—figure supplement 1C). We assembled bispecific IgGs and diabody-Fc fusions, in which one arm targeted FZD and the second arm targeted one of the Wnt binding sites on LRP6. None of these Abs were able to activate βcatenin signaling, but rather, they all acted as Wnt signaling antagonists (Figure 1—figure supplement 1D–E). We thus concluded that bivalent recruitment of FZD/LRP6 heterodimers by bispecific Abs, using these configurations, failed to recapitulate the stoichiometric, geometric or conformational properties of FZD/LRP6 complexes induced by natural Wnt ligands to stimulate signaling.

Recent reports showed that Wnt induces dimerization of FZD CRD (Hirai et al., 2019; Nile et al., 2017) and that FZD and LRP6 clustering may be required for signaling (Carron et al., 2003; Chen et al., 2014; Gong et al., 2010; Hua et al., 2018). Thus, we hypothesized that polyvalent binding to FZD and LRP6 may be required for effective signaling, and we developed a novel tetravalent antibody format to explore this concept (Figure 1C and Figure 1—figure supplement 1F-H). The format consisted of a homodimeric or heterodimeric Fc with a diabody at either end, and thus contained four Ab paratopes that could be engineered individually for any specificity. We built three tetravalent molecules that all contained two pan-FZD paratopes at the N-termini of the Fc but differed in the nature of the anti-LRP6 paratopes at their C-termini: one targeted two WNT1 binding sites (F^P+P^-L6^1+1^), another targeted two WNT3A binding sites (F^P+P^-L6^3+3^), and the third contained one paratope each for the WNT1 and WNT3A binding sites (F^P+P^-L6^1+3^). F^P+P^-L6^3+3^ and F^P+P^-L6^1+1^ both activated βcatenin signaling but the former was ~10 fold more efficacious (Figure 1D). Somewhat surprisingly, F^P+P^-L6^1+3^ was~2 fold more efficacious than even F^P+P^-L6^3+3^, indicating that engagement of a strong WNT3A site and a weak WNT1 site together is more effective than engagement of two strong WNT3A sites. The two best FLAgs had single-digit nanomolar potency (EC_50_ ~5 nM), which was virtually identical to the potency of purified WNT3A, and displayed a bell-shaped dose response profile (Figure 1—figure supplement 1I–J). We interpret this as indicating that maximal stimulation requires multivalent binding of the FLAg and that decreased efficacy at higher concentrations is likely attributable to monovalent binding to either FZD or LRP6. Treatment of RKO cells, which express low levels of βcatenin (Major et al., 2007), with F^P+P^-L6^1+3^ caused dose- and time-dependent increases in βcatenin protein levels and phosphorylation of DVL2, a hallmark of Wnt-FZD pathway activation (Figure 1E and F). Thus, tetravalent FLAgs are modular, engineerable, human Ab modalities that function as synthetic agonists of FZD and LRP6.

### Characterization and dissection of FLAg binding and activity

**Figure 2.**
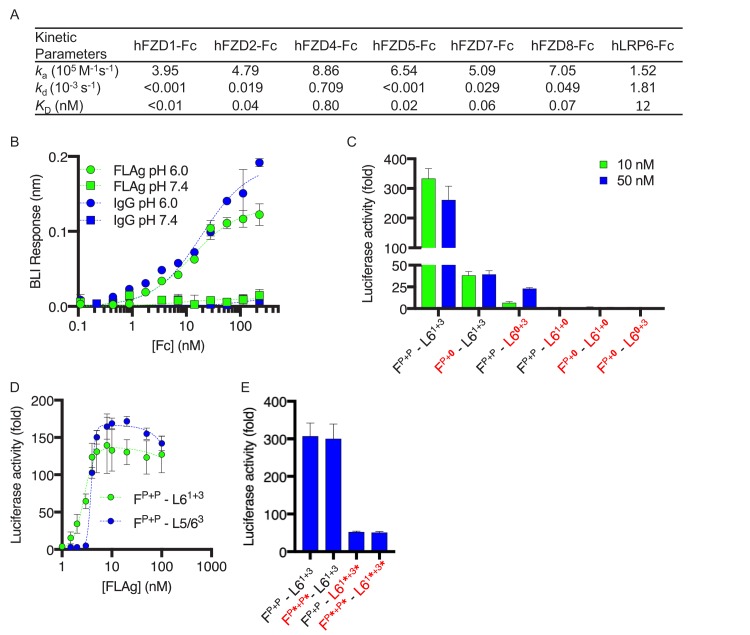
Characterization and structure-function activity relationships for FLAg F^P+P^-L6^1+3^. (**A**) BLI measurements of kinetic parameters for F^P+P^-L6^1+3^ binding to immobilized, Fc-tagged FZD CRDs and LRP6 ECD (**B**) Dose response curves for BLI measurements of F^P+P^-L6^3+3^ and IgG binding to immobilized FcRn at pH 6 or 7.4. Error bars indicate SD, n = 2. (**C**) Agonist activities of F^P+P^-L6^1+3^ variants with disabled paratopes. ‘0’ indicates substitution with an anti-MBP paratope. HEK293T cells expressing a LEF/TCF reporter gene were stimulated with 10 or 50 nM of the indicated FLAg, and the fold increase in luciferase activity was determined relative to an unstimulated control. Error bars indicate SEM, n = 3. (**D**) Activation of βcatenin signaling by FLAgs. Dose response curves are shown for the activation of a LEF/TCF reporter gene (*y-axis*) by serial dilutions of indicated FLAgs (*x-axis*). Error bars indicate SEM, n = 3. (**E**) Agonist activities of F^P+P^-L6^1+3^ variants with scFvs in place of diabodies. Asterisks (*) indicate scFvs. Assays were performed as in (**C**). Error bars indicate SEM, n = 3. 10.7554/eLife.46134.008Figure 2—source data 1.Source data for Figure 2B. 10.7554/eLife.46134.009Figure 2—source data 2.Source data for Figure 2C. 10.7554/eLife.46134.010Figure 2—source data 3.Source data for Figure 2D. 10.7554/eLife.46134.011Figure 2—source data 4.Source data for Figure 2E.

To confirm the engineered affinity and specificity of the optimal FLAg F^P+P^-L6^1+3^, we used Bio-Layer Interferometry (BLI) to measure its binding kinetics to nine of the 10 human FZD CRDs and to human LRP6 ECD (Figure 2A and Figure 1—figure supplement 1A). As anticipated, the FLAg bound with affinities in the picomolar range (*K*_D_ = 10–800 pM) to the six FZDs recognized by the FZD diabodies derived from the parent pan-FZD paratope (Figure 1A) (Pavlovic et al., 2018) but did not bind detectably to the other three FZDs. Moreover, affinity for LRP6 was in the nanomolar range (*K*_D_ = 12 nM). We then used BLI to assess FLAg binding to various Fc receptors. The FLAg behaved similarly to a conventional IgG and interacted with FcRn in a dose and pH dependent manner (Figure 2B). Natural IgGs bind to FcRn at pH 6.0 but not at pH 7.4, and this enables recycling during pinocytosis and consequent long half-life in vivo. The FLAg also behaved similarly to the IgG for interaction with other Fc effectors including complement (C1q), the natural killer cell marker CD16a, the B cell marker CD32a, and the monocyte and macrophage marker CD64 (Figure 2—figure supplement 1B). We conclude that the FLAg contains a functional Fc moiety that should confer effector functions and long half-life in vivo.

**Figure 2—figure supplement 1.**
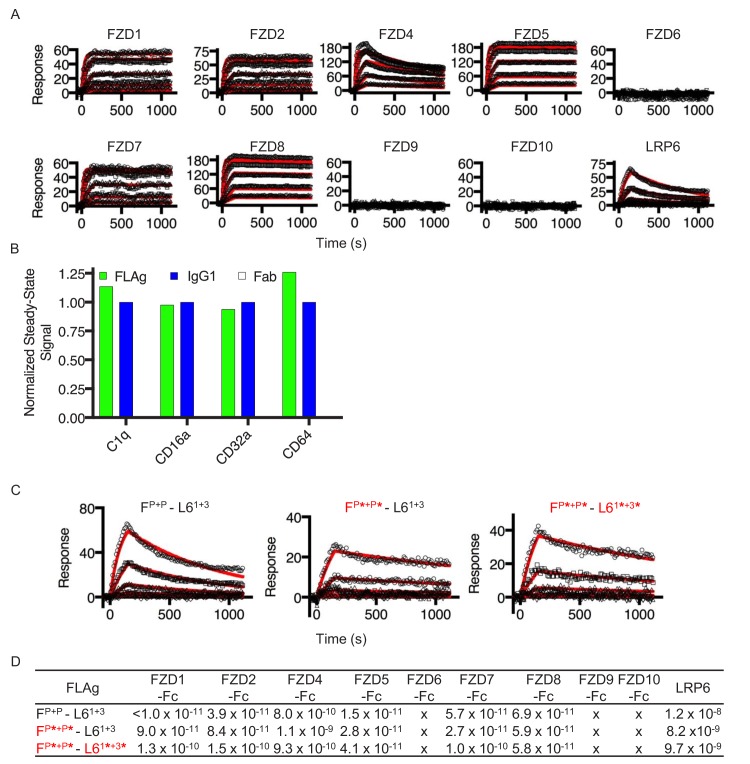
Biophysical properties of FLAgs. (**A**) Surface plasmon resonance sensograms of FZD recognition by F^P+P^ - L6^1+3^ at concentrations ranging from 100nM- 1.23pM. (**B**) BLI steady-state measurements for FLAg, IgG and a negative control Fab binding to immobilized mediators of immune effector functions. Signals for FLAg were normalized to the IgG and Fab binding controls, n = 1. (**C**) Surface plasmon resonance sensograms of LRP6 recognition for the indicated FLAg at concentrations ranging from 100 nM to 1.23 nM. (**D**) Binding kinetics table of the FLAg constrained as scFv or diabodies to the various FZD isoforms.

The modular design of the tetravalent F^P+P^-L6^1+3^ FLAg allowed us to dissect the contributions of each of the four paratopes to the intrinsic agonist activity by replacing each with a null paratope binding to the irrelevant antigen maltose-binding protein (MBP). βcatenin signaling assays showed that maximal stimulation was reduced significantly by disabling one anti-FZD paratope or the anti-LRP6 paratope for the WNT1 binding site and was completely ablated by disabling the anti-LRP6 paratope for the WNT3A binding site or by simultaneously disabling one anti-FZD paratope and either of the anti-LRP6 paratopes (Figure 2C). We also substituted an anti-LRP5 paratope targeting the WNT3A binding site for the anti-LRP6 paratope targeting the WNT1 binding site to generate a molecule (F^P+P^-L5/6^3^) that could recruit both co-receptors and observed activity similar to that of F^P+P^-L6^1+3^ (Figure 2D, EC_50_ = 4 nM). Taken together, these data showed that optimal agonist activity is achieved with a molecule capable of recruiting two FZDs through a common epitope and LRP6 through two distinct epitopes, but activity can be modulated to intermediate levels by disabling one of the anti-FZD or anti-LRP6 paratopes. Moreover, molecules that could recruit FZD and two different co-receptors were generated by combining two anti-FZD paratopes with one paratope each for LRP5 and LRP6.

We also explored the requirements for geometric and spatial constraints imposed by the intermolecular diabody format by substituting diabody pairs with pairs of less constrained intramolecular single-chain variable fragments (scFvs) (Figure 2E). Compared with F^P+P^-L6^1+3^, a FLAg that contained anti-FZD scFvs (F^P*+P*^-L6^1+3^) exhibited similar activity, whereas activity was significantly reduced for FLAgs that contained anti-LRP6 scFvs (F^P+P^-L6^1*+3*^) or scFvs at both ends (F^P*+P*^-L6^1*+3*^). These differences in activity were not due to differences in affinity, as BLI measurements showed comparable, high-affinity binding to LRP6 and FZD isoforms regardless of whether paratopes were presented in the diabody or scFv format (Figure 2—figure supplement 1C–D). Taken together, these results showed that particular stoichiometries and geometries are required for the assembly of optimal FZD/LRP6 signaling complexes, and constraints are especially precise for LRP6, which requires engagement of two distinct epitopes in a specific geometry dictated by the diabody format. Notably, the looser constraints for FZD engagement enabled significant activation with a single anti-FZD paratope (Figure 2C), which opens the door for further enhancing specificity or altering signaling by recruiting a different cell surface protein through an additional paratope in conjunction with an anti-FZD paratope at the N-termini of the heterodimeric Fc.

### Assembly and activity of FLAgs with tailored specificities

**Figure 3.**
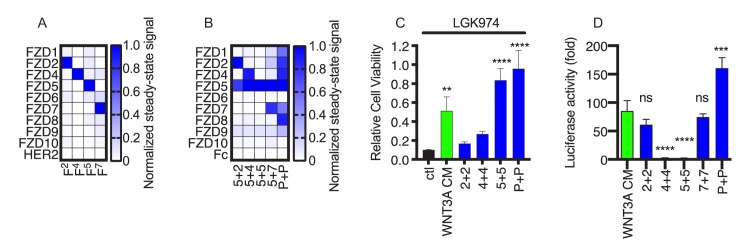
FLAgs with tailored specificities and activities. (**A**) Anti-FZD Fab specificity. The heat map represents the steady state BLI signal for 100 nM Fab (*x-axis*) binding to immobilized, Fc-tagged FZD CRD or negative control HER2 ECD (*y-axis*), normalized to the highest signal. (**B**) Specificity of FLAgs for FZD CRDs. For each FLAg (*x-axis*) of the form F^x+y^-L6^1+3^, where ‘x’ and ‘y’ are paratopes from monospecific Fabs shown in (**A**), the heat map represents the normalized steady state BLI signal, as described in (**A**). (**C**) Effects of FLAgs on the viability of HPAF-II cells treated with the Porcupine inhibitor LGK974. Cells were treated with 100 nM LGK974 alone (ctl), or along with WNT3A conditioned media (WNT3A) or 100 nM FLAg of the form F^x+x^-L6^1+3^ (‘x + x’ indicated on the *x-axis*) with specificities shown in (**C**). Endpoint proliferation was measured by quantification of crystal violet staining and cell viability was normalized to the viability of cells that were not treated with LGK974. N = 3 independent experiments. One-way ANOVA with Dunnett’s multiple comparison test with control treated. **p<0.01, ****p<0.0001. (**D**) Delineation of FLAg specificity for βcatenin mediated signaling in HEK293 cells. Cells stably expressing a LEF/TCF luciferase reporter were stimulated with various FLAgs specific for individual Frizzled receptors. FZD2 (F^2+2^-L6^1+3^) and FZD7 (F^7+7^-L6^1+3^) specific FLAgs as well as the pan-FLAg (F^P+P^-L6^1+3^) robustly stimulate βcatenin signaling whereas FZD4 and FZD5 specific FLAgs are inactive in this context. Error bars indicate SEM, n = 5. One-way ANOVA with Dunnett’s multiple comparison test with control treated. ****p<0.0001, ***p=0.0003, ns = not significant. 10.7554/eLife.46134.014Figure 3—source data 1.Source data for Figure 3A. 10.7554/eLife.46134.015Figure 3—source data 2.Source data for Figure 3B. 10.7554/eLife.46134.016Figure 3—source data 3.Source data for Figure 3C. 10.7554/eLife.46134.017Figure 3—source data 4.Source data for Figure 3D.

We next investigated whether highly specific anti-FZD Abs could be used as building blocks to assemble FLAgs with tailored specificities. To explore this concept, we took advantage of four monospecific anti-FZD Abs (Figure 3A and Figure 3—figure supplement 1A) and built a panel of FLAgs, each of which contained the anti-FZD5 paratope and a second monospecific paratope. A FLAg containing two identical anti-FZD5 paratopes exhibited monospecificity for FZD5. Moreover, when paired with one of the other monospecific anti-FZD paratopes, the anti-FZD5 paratope acted in an additive manner, enabling the facile assembly of bispecific FLAgs in a predictable manner (Figure 3B). Importantly, bispecific FLAgs maintain high affinities for the respective targeted Frizzled (Figure 3—figure supplement 1B and 1C). Recent genetic and functional data support the idea that each Wnt protein may be endowed with a particular function determined by its FZD specificity profile (Dijksterhuis et al., 2015; Voloshanenko et al., 2017), and thus, FLAgs that mimic the specificities of particular Wnts would be useful analogs of those Wnt isoforms. We have shown that it is possible to engineer anti-FZD Abs covering numerous specificity profiles ranging from monospecific to pan-specific. Thus, given the modular and the additive nature of the FLAg architecture, we anticipate that it will be possible to build synthetic agonists with diverse and tailored specificities, including mimics of known Wnt specificity profiles.

**Figure 3—figure supplement 1.**
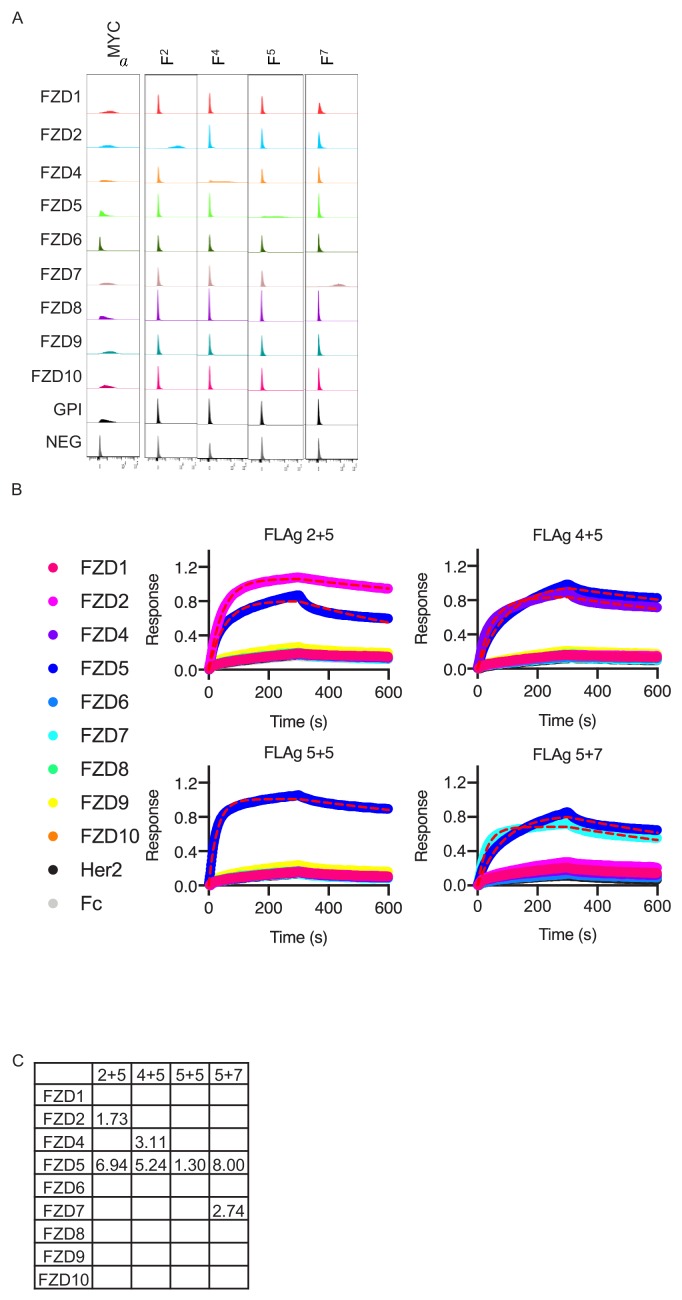
Selectivity of Fabs for FZD CRD expressed on cells. (**A**) Flow cytometry specificity analysis of selective FZD Fabs (Steinhart et al., 2017). The specificity of each Fab was determined by measuring binding to a panel of recombinant CHO lines displaying each FZD ECD at the cell surface using a GPI anchor (Myc-FZD ECD-GPI). Cells were stained with 10 nM of each Fab and binding evaluated using flow cytometry. (**B**) Raw chromatograms of FLAg specificity by BLI. (**C**) Table of extrapolated *K*_D_ (nM) from locally fit kinetics of each FLAg to human FZD ECDs using a 1:1 Langmuir model.

To assess the activity of the FZD5-specific FLAg, we used *RNF43* mutant cancer cells in which Wnt-βcatenin signaling mediated through FZD5 is essential for proliferation (Steinhart et al., 2017). Treatment of *RNF43* mutant pancreatic adenocarcinoma cell line HPAF-II with LGK974, a small molecule that inhibits all Wnt secretion, led to cell cycle arrest and potent inhibition of proliferation (Figure 3C). Cell proliferation was partially rescued by WNT3A conditioned media and was fully rescued by a pan-FZD or FZD5-specific FLAg, which is consistent with these cells requiring signaling through FZD5 (Figure 3C). Consistent with genetic data (Voloshanenko et al., 2017) showing that FZDs 1, 2 and 7 are required for Wnt-mediated βcatenin activation in HEK293 cells, FLAgs specific for FZD2 or FZD7 activated βcatenin robustly in these cells whereas FLAgs specific for FZD4 or FZD5 did not (Figure 3D). Taken together, these results show that our diverse anti-FZD Abs can be used as modular building blocks to assemble FLAgs with tailored specificities in a predictable manner. Furthermore, we envision that tailored FLAgs will enable precise mimicry of natural Wnts and will provide novel possibilities for basic research and therapeutic applications.

### Phenotypic effects of FLAgs in cells, organoids and animals

**Figure 4.**
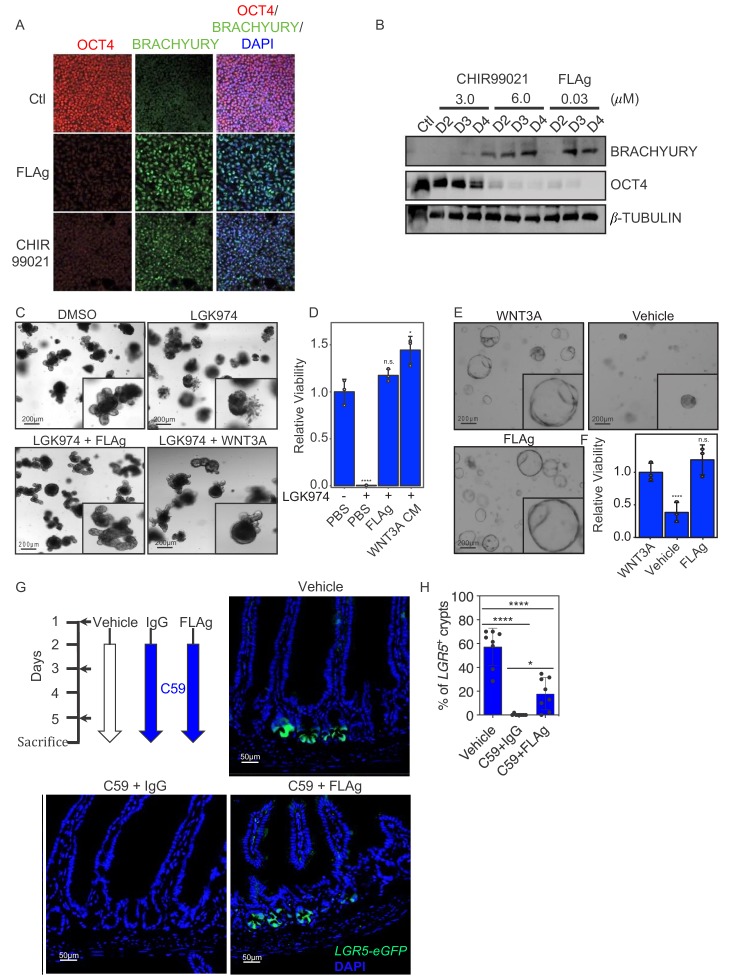
FLAg activity in cells, organoids and animals. (**A,B**) Mesoderm differentiation of hPSCs induced by FLAg or the GSK3 inhibitor CHIR99021. Human PSCs were treated with 30 nM pan-FLAg (F^P+P^-L6^1+3^) or with 3 or 6 μM CHIR99021 for the indicated times. Cells were fixed and visualized by immunofluorescence (**A**) or lyzed and analyzed by western blotting (**B**) to assess the levels of the pluripotency marker OCT4 and the early mesodermal lineage marker BRACHYURY. Immunofluorescence staining and western blots are representative of three and two independent experiments respectively. (**C**) Representative images of mouse small intestinal organoids with indicated treatments (1 μM LGK974, 1 μM LGK974 +30 nM pan-FLAg, 1 μM LGK974 +50% WNT3A conditioned media) (**D**) Viability of mouse small intestinal organoids following indicated treatments. Bars represent mean fold change ±s.d., representative of n = 3 independent experiments. Statistical analysis was performed by one-way ANOVA Dunnett’s test *p≤0.05, ****p≤0.0001. (**E**) Culture of human colon organoids with indicated treatments (10 nM pan-FLAg, control conditioned media or 50% WNT3A conditioned media). (**F**) Viability of human colon organoids following indicated treatments. Bars represent mean fold change ±s.d., representative of n = 3 independent experiments. Statistical analysis was performed by one-way ANOVA Dunnett’s test ****p≤0.0001. (**G**) Schematic of the in vivo workflow to evaluate FLAg efficacy. Vehicle (PBS), control IgG (10 mg/kg i.p) or FLAg (10 mg/kg i.p) groups were injected on day 1,3 and 5. The IgG and FLAg groups were further administered C59 (50 mg/kg, oral gavage) twice daily starting on day2. Representative fluorescence images of small intestinal sections from *LGR5*-GFP mice treated with Vehicle, IgG +C59 or pan-FLAg +C59. *LGR5*-GFP (green) is expressed in the stem cells at the bottom of crypts. Cell nuclei were counterstained with DAPI. (**H**) Quantification of LGR5 +crypts. Percentage of LGR5 +crypts was evaluated by counting at least 100 crypts per mouse in four different mice in two independent experiments (eight mice total per group). Statistics, one way ANOVA followed by Tukey test compared to vehicle treated group. *p<0.05, ****p<0.0001. 10.7554/eLife.46134.019Figure 4—source data 1.Source data for Figure 4D. 10.7554/eLife.46134.020Figure 4—source data 2.Source data for Figure 4E. 10.7554/eLife.46134.021Figure 4—source data 3.Source data for Figure 4H.

Having established that FLAgs selectively engage FZD and LRP to activate Wnt-associated signaling pathways, we explored the phenotypic effects of these signals in progenitor stem cells (PSCs), organoids and animals. Modulation of Wnt-βcatenin signaling activity is integral to most PSCs differentiation protocols (Huggins et al., 2016). Treatment of human PSCs with WNT3A conditioned media, or small molecule inhibitors of GSK3, activates βcatenin signaling, leads to primitive streak induction, and promotes mesodermal fate specification (Davidson et al., 2012). We evaluated FLAg activity in this context and found that treatment with 30 nM F^P+P^-L6^1+3^ for three days caused robust induction of the mesoderm marker BRACHYURY and decreased expression of the pluripotency marker OCT4 to levels comparable to treatment with the GSK3 inhibitor CHIR99021 at 6 μM (Figure 4A and B).

Being pervasive stem cell niche factors, Wnts and R-spondins are required for the derivatization and maintenance of three-dimensional culture organoids from many tissues. In vitro, Wnt proteins secreted by paneth cells are sufficient to support the growth of mouse small intestine organoids in the presence of R-spondins. To assess whether a FLAg could substitute for endogenous Wnts, we treated the organoids with LGK974, which ablated Wnt production and inhibited growth completely, and asked whether F^P+P^-L6^1+3^ could rescue growth. Addition of either WNT3A conditioned media or F^P+P^-L6^1+3^ completely rescued the growth defects caused by LGK974 (Figure 4C and D). Contrary to mouse small intestine organoids, human colorectal organoids do not have paneth cells and thus require exogenous source of Wnt growth factors. Incorporation of WNT3A conditioned media or F^P+P^-L6^1+3^ was sufficient to propagate these organoids for multiple passages (Figure 4E and F). We conclude that a pan-FZD FLAg can substitute for Wnt proteins to support growth of tissue organoids, and thus, the FLAg is a defined component that could alleviate limitations associated with the use of conditioned media or purified Wnt proteins.

F^P+P^-L6^1+3^ recognizes mouse FZDs and LRP6, and it contains an Fc that interacts with the FcRn and should endow it with a long, Ab-like, half-life in vivo. Thus, we tested in mice whether F^P+P^-L6^1+3^ could accumulate to levels that would be sufficient to activate βcatenin signaling and mobilize endogenous stem cell activity. Within the intestinal stem cell niche, Wnt proteins secreted by mesenchymal cells induce expression of βcatenin target genes in stem cells at the bottom of the crypt to direct their self-renewal, and the target gene *LGR5* is frequently used as a marker of stem cells in various tissues. Treatment of *LGR5*-GFP mice with C59 ablated Wnt production and caused rapid extinction of *LGR5* expression and the linked GFP signal in crypt stem cells. Strikingly, GFP expression was rescued upon co-treatment with F^P+P^-L6^1+3^ by intraperitoneal injection (Figure 4G and H). We conclude that F^P+P^-L6^1+3^ has a sufficient half-life and bioavailability to enable βcatenin activation at levels that promote self-renewal of intestinal stem cells in the absence of endogenous Wnt.

## Discussion

Modular tetravalent agonists for tailored activation of FZD and its co-receptors will enable precise interrogation and control of Wnt signaling circuits in vitro and in vivo. Indeed, full control over specificities, achieved by rationally combining paratopes within the four targeting sites of FLAgs, makes possible the functional mimicry of the signaling properties intrinsic to each Wnt. Moreover, monospecific and bispecific FLAgs can be engineered to activate signals with greater precision than is possible with natural Wnts. Because significant activity can be achieved with only one FZD binding site, the fourth site within the tetravalent FLAg represents an opportunity for additional engineering with tissue-specific paratopes to confer localized activity. We also envision that paratopes for other Wnt co-receptors, such as ROR1/2, RYK and PTK7, may be used to build FZD agonists to activate alternate βcatenin-independent Wnt pathways that remain under-explored due to a lack of specific tools.

In terms of mechanism of action, the requirement for tetravalency in optimal FLAgs likely indicates that maximal activity requires assembly of FZD and LRP5/6 in higher order complexes. Also, the fact that F^P+P^-L6^1+3^ was more active than F^P+P^-L6^3+3^ suggests that engagement of both Wnt binding sites within LRP6 may result in intramolecular rearrangements that stabilize conformations that are more favorable for signaling activity. Intriguingly, recent structural work reported a 2:2 stoichiometry of Wnt ligand and FZD CRD that could be modeled to recruit two LRP5/6 molecules (Hirai et al., 2019). Our results suggest that bivalent recruitment of one FZD and one LRP5/6 molecule is sufficient for signaling activity but engagement of an oligomeric complex, composed of two FZD and one or two LRP5/6 molecules, is required for maximal activation. Therefore, tetravalent FLAg molecules may be better than bivalent agonists for recapitulating mechanisms employed by Wnt proteins to engage FZD-LRP5/6 receptor complexes. Additional work to probe the structural basis for FLAg activity may shed light on the molecular mechanisms underlying Wnt receptor activation.

Practically, we foresee that the defined composition, high specific activity, and modular design of FLAgs will aid the derivatization and maintenance of three-dimensional tissue organoids and will profoundly impact the directed differentiation of progenitor stem cells, paving the way for disease modeling and cell therapy. Ultimately, the Ab-like character and bioavailability of FLAgs opens up new therapeutic possibilities for the mobilization of stem cells to promote tissue regeneration and to activate FZD complexes in human disease contexts where their activity is deficient.

## Materials and methods

### Ab selections and screens

The phage-displayed synthetic library F was used to select for Fabs that bound to Wnt receptors, as described (Persson et al., 2013). Briefly, Fc-tagged ECD protein (R and D Systems) was immobilized on Maxisorp immunoplates (ThermoFisher, catalog number 12-565-135) and used for positive binding selections with library phage pools that were first exposed to similarly immobilized Fc protein to deplete non-specific binders. After 4 rounds of binding selections, clonal phage were prepared and evaluated by phage ELISA (Birtalan et al., 2008). Clones that displayed at least 10-fold greater signal for binding to antigen compared with Fc were considered to be specific binders that were subjected to further characterization.

### Recombinant proteins and reagents

Fc-tagged fusions of FZD1 (5988-FZ-050), FZD2 (1307-FZ-050), FZD4 (5847-FZ-050), FZD5 (1617-FZ-050), FZD7 (6178-FZ-050), FZD8 (6129-FZ-050), FZD9 (9175-FZ-050), FZD10 (3459-FZ-050) were purchased from R and D Systems. The Fc-tagged ECD of FZD6 (residues 19–132, Uniprot O60353-1) was expressed and purified from Expi293 cells using the pFUSE-hIgG1-Fc2 vector (invivogen) and the single protomer species was separated from aggregated protein by size exclusion chromatography on a Superdex 200 (10/300) column (GE Healthcare). Fc-tagged ECD fusion proteins of human (1505-LR-025) and mouse (2960-LR-025) LRP6 and mouse LRP5 (7344-LR-025/CF) were purchased from R and D Systems. WNT1 (SRP4754-10ug), WNT2b (3900-WN-010/CF), WNT5a (645-WN-010/CF) and WNT3A (5036-WN-010/CF) were purchased from R and D Systems, and WNT3A conditioned media was prepared as described (Lui et al., 2011). Other proteins and chemicals were purchased from the following suppliers: FcRN (R and D, 8693-FC), C1q (Sigma, C1740), CD16a (R and D, 4325-FC), CD32a (R and D, 1330 CD/CF), CD64 (R and D, 1257-FC), LGK974 (Cayman Chemicals), C59 (Dalriada Therapeutics), and CHIR99021 (Sigma Aldrich).

### FLAg and Ab cloning

DNA fragments encoding Ab variable domains were either amplified by the PCR from phagemid DNA template or were constructed by chemical synthesis (Twist Biosciences). The DNA fragments were cloned into in-house mammalian expression vectors (pSCSTa) designed for production of kappa light chains and human IgG1 heavy chains. Bispecific diabodies and IgGs contained an optimized version of a ‘knobs-in-holes’ heterodimeric Fc (Ridgway et al., 1996; Van Dyk et al., 2018). FLAg and diabody-Fc fusions were arranged a VH-VL orientation with the variable domains separated by a short GGGGS linker, which favors intermolecular association between VH and VL domains and thus favors diabody formation. To produce diabody-Fc fusion constructs, diabody chains were fused to human IgG1 Fc. FLAg proteins were constructed as VH-*x*-VL-*y*-[human IgG1 Fc]-*z*-VH-*x*-VL where linkers are *x* = GGGGS, *y* = LEDKTHTKVEPKSS, and *z* = SGSETPGTSESATPESGGG. In this format, the human IgG1 Fc or knob-in-hole IgG1 Fc fragments spanned from position 234–478 (Kabat numbering). For scFv-Fc fusions, the variable domains were arranged in a VL-VH orientation and were connected by a long GTTAASGSSGGSSSGA linker, which favors intramolecular association between VH and VL domains and thus favors scFv formation. For all constructs, the entire coding region was cloned into a mammalian expression vector in frame with the secretion signal peptide.

### Protein expression and purification

Antigen, Ab and FLAg proteins were produced in Expi293F (ThermoFisher) cells by transient transfection. Briefly, cells were grown to a density of approximately 2.5 × 10^6^ cells/ml in Expi293 Expression Media (Gibco) in baffled cell culture flasks and transfected with the appropriate vectors using FectoPRO transfection reagent (Polyplus-transfection) using standard manufacturer protocols (ThermoFisher). Expression was allowed to proceed for 5 days at 37°C and 8% CO_2_ with shaking at 125 rpm. After expression, cells were removed by centrifugation and protein was purified from the conditioned media using rProtein A Sepharose (GE Healthcare). Purified protein was buffer exchanged into either PBS or a formulated stabilization buffer (36.8 mM citric acid, 63.2 mM Na_2_HPO_4_, 10% trehalose, 0.2 M L-arginine, 0.01% Tween-80, pH 6.0) for storage. Protein concentrations were determined by absorbance at 280 nm and purity was confirmed by SDS-PAGE analysis.

### In vitro binding assays

BLI assays were performed using an Octet HTX instrument (ForteBio). For measuring binding to antigen, FZD-Fc proteins were captured on AHQ BLI sensors (18–5001, ForteBio) to achieve a BLI response of 0.6–1 nm and remaining Fc-binding sites were saturated with human Fc (009-000-008, Jackson ImmunoResearch). FZD-coated or control (Fc-coated) sensors were transferred into 100 nM Ab or FLAg in assay buffer (PBS, 1% BSA, 0.05% Tween20) and association was monitored for 300 s. Sensors were then transferred into assay buffer and dissociation was monitored for an additional 300 s. Shake speed was 1000 rpm and temperature was 25°C. End-point response values were taken after 295 s of association time. End-point data were analyzed by subtracting the Fc signal from the FZD-Fc signal and then normalizing the data to the highest binding signal.

For measuring binding to Fc receptors, Abs or FLAgs were immobilized on AR2G sensors (18–5092, ForteBio) by amine coupling to achieve a BLI response of 0.6–3 nm and remaining sites were quenched with ethanolamine. Coated sensors were equilibrated in assay buffer (PBS, 1% BSA, 0.05% Tween20) and transferred into Fc receptor solutions. Association was monitored for 600 s, the sensors were transferred to assay buffer, and dissociation was monitored for 600 s. CD64 and all other Fc receptors were assayed at 50 nM or 300 nM, respectively, at pH 7.4, unless as indicated. Shake speed was 1000 rpm and temperature was 25°C. End-point response values were taken at the end of the association phase and were normalized to isotype controls. Steady-state FcRN binding assays were performed in a similar manner, except that FcRN was immobilized and serial dilutions (0.1–225 nM) of Ab or FLAg were assessed in solution. The association and dissociation times were 600 or 1200 s, respectively.

Surface plasmon resonance (SPR) assays were performed using a ProteOn XPR36 system (Bio-Rad). FZD-Fc or LRP-Fc proteins were immobilized to GLC sensor surface (176–5011) using standard amine coupling chemistry. Abs or FLAgs in assay buffer (PBS, 0.05% Tween20, 0.5% BSA) were injected at 40 μl/min and association was monitored for 150 s. Assay buffer was then injected at 100 μl/min and dissociation was monitored for 900 s. Assays were performed at 25°C. Analysis was performed using a 1:1 Langmuir model and globally fit to determine k_on_ and k_off_ values using ProteOn Manager software. K_D_ was calculated as the ratio of k_off_/k_on_.

### Epitope binning

BLI epitope binning experiments were performed using an Octet HTX instrument (ForteBio). FZD-Fc or LRP6-Fc protein was immobilized on AHQ (18–5001, ForteBio) or AR2G (18–5092, ForteBio) BLI sensors, respectively. Coated sensors were transferred into 100 nM Ab in assay buffer (PBS, 1% BSA, 0.05% Tween20) for 240 s to achieve saturation of binding sites. Sensors were then transferred into 100 nM competing Ab in assay buffer for 180 s. Response at 20 s after exposure to competing Ab was measured and normalized to binding signal on unblocked antigen-coated sensors. Shake speed was 1000 rpm and temperature was 25°C.

### Cell culture

HPAF-II and HEK293T cell lines were maintained in DMEM containing 4.5 g/L D-glucose, Sodium pyruvate, L-glutamine (ThermoFisher #12430–054) and supplemented with 10% FBS (ThermoFisher) and Penicillin/Streptomycin (ThermoFisher #15140–163). CHO cells were maintained in DMEM/F12 (ThermoFisher #11320–033) supplemented with 10% FBS and penicillin/streptomycin. Cells were maintained at 37°C and 5% CO_2_.

H1 hESCs were cultured on Geltrex-coated (1:100 in DMEM/F12; Gibco) plates and maintained in StemFlex basal medium (Gibco) supplemented with 1% Pen-Strep (Gibco). For cell passage, cells were washed once with PBS and dissociated using TrypLE Select enzyme (Gibco) and neutralizing STOP solution (10% FBS in DMEM/F12). Cells were then plated onto Geltrex-coated plates in StemFlex supplemented with RevitaCell (100x; Gibco). RevitaCell was removed the next day and media was changed every 2 days. For cell differentiation experiments, cells were treated with CHIR99021 (3–6 μM) or pan-FLAg F^P+P^-L^1+3^ (30nM) for 3 days in StemFlex. All differentiations were conducted on G-banded karyotyped H1s grown in feeder-free and monolayer conditions in StemFlex.

### Flow cytometry

Indirect immunofluorescence staining of cells was performed with 10 nM anti-FZD Fab for the CHO cell lines as previously described (Steinhart et al., 2017). Alexa Fluor 488 AffiniPure F(ab′)two was used as the secondary antibody (Jackson ImmunoResearch, 109-545-097). Anti-c-Myc IgG1 9E10 (primary antibody, ThermoFisher, MA1-980) and Alexa Fluor 488 IgG (secondary antibody, Life technologies, A11001) were used as negative controls. All reagents were used as per manufacturer's instructions.

### Luciferase reporter assay

HEK293T cells were transduced with lentivirus coding for the pBARls reporter (Biechele and Moon, 2008) and with Renilla Luciferase as a control to generate a Wnt-βcatenin signaling reporter cell line. 1–2 × 10^3^ cells in 120 µl were seeded in each well of 96-well plates for 24 hr prior to transfection or stimulation. The following day, FLAg or Ab protein was added, and following 15–20 hr of stimulation, cells were lysed and luminescence was measured in accordance with the dual luciferase protocol (Promega) using an Envision plate reader (PerkinElmer).

### Western blot assay

H1 ESCs were solubilized with lysis buffer (1% Nonidet P-40, 0.1% sodium dodecyl sulfate (SDS), 0.1% deoxycholic acid, 50 mM Tris (pH 7.4), 0.1 mM EGTA, 0.1 mM EDTA, 20 mM sodium fluoride (NaF), 1:500 protease inhibitors (Sigma) and 1 mM sodium orthovanadate (Na_3_VO_4_)). Lysate was incubated for 30 min at 4°C, centrifuged at 14,000 × *g* for 10 min, boiled in SDS sample buffer, separated by SDS-polyacrylamide gel electrophoresis, transferred onto a nitrocellulose membrane and Western blotted using indicated Abs. Ab detection was performed by a chemiluminescence-based detection system (ECL; ThermoFisher).

### Crystal violet proliferation assay

HPAF-II cells were seeded at 500 cells per well, and after 24 hr, 100 nM LGK974 was added with or without 100 nM FLAg. Medium was changed and drug treatment was renewed every other day. Cells were fixed with ice-cold methanol after 7 days treatment. Cells were stained with 0.5% crystal violet solution in 25% methanol, destained in 10% acetic acid and quantified by measuring absorbance at 590 nm.

### Immunofluorescence

H1 hESCs treated with FLAg and CHIR99021 for 3 days were washed with cold PBS, and fixed for 20 min with 4% PFA. Fixed cells were rinsed with PBS, permeabilized with 0.3% triton for 10 min, and blocked with 1% BSA for 1 hr. Cells were incubated for 2 hr with primary Abs for BRACHYURY (R and D systems AF2085; goat; dilution 1:100) or OCT3/4 (Santa Cruz sc5279; mouse; dilution 1:100) in 1% BSA and 1 hr with Alexa Fluor 488-labeled donkey anti-goat or Alexa Fluor 568-labeled donkey anti-mouse Ab. Coverslips were mounted using Fluoromount (Sigma-Aldrich) and analyzed on a Zeiss LSM700 confocal microscope using a 60 × oil objective. Images were assembled using ImageJ and Photoshop CS6 (Adobe Systems, Mountain View, CA).

### Organoid assay

An 8 week old, female, C57BL/6 mouse was sacrificed and small intestine crypts were harvested for organoid isolation and cultured as previously described (O'Rourke et al., 2016). Human colon organoids were cultured as previously described (Sato et al., 2011). Organoid cultures were passaged and embedded in 25 μl Growth Factor Reduced Matrigel (Corning, 356231) and plated in triplicates in a 48-well plate. Indicated treatments (small intestine: vehicle, 1 μM LGK974, 1 μM LGK974 +50% WNT3A conditioned media, 1 μM LGK974 +30 nM FLAg; colon: WNT3A conditioned media, removal of Wnt from media, 10 nM FLAg) were added to 250 μl of complete media, added to each well on day of passaging and changed every 2–3 days.

At the endpoint (small intestine: 7 days; colon: 12 days), 150 μl CellTiter-Glo3D (Promega) was added to 150 μl media in each well. Organoids were lysed on a rocking platform for 30 min at room temperature. The luminescence reading was measured in duplicates for 20 μl lysate from each well on the Envision multilabel plate reader. The average luminescence reading for each condition was normalized to the control condition to calculate viability.

### Activation of LGR5 intestinal stem cells in mouse intestinal crypts

8–10 week-old Lgr5-EGFP-IRES-creERT2 (B6.129P2-*Lgr5^tm1(cre/ERT2)Cle^*/J) mice were purchased from The Jackson Laboratory (Bar Harbor, ME). All experiments were performed according to protocols approved by the Animal Care and Use Committee at the University of Toronto, and complied with the regulations of the Canadian Council on Animal Care and with the ARRIVE guidelines (Animal Research: Reporting in vivo Experiments). F^P+P^-L6^1+3^ or a negative control Ab was reconstituted in 37 mM Citric Acid, 63 mM Na2HPO4, 10% trehalose, 0.2M L-Arginine, 0.01% polysorbate 80, pH 6.0. The Porcupine Inhibitor C59 was reconstituted with 0.5% methylcellulose mixed with 0.1% Tween 80 in ddH2O. The mice (male and female) were divided into three groups (4–6 per group): vehicle, IgG (C59 +control Ab) or FLAg (C59 + F^P+P^-L6^1+3^). On day 1, 3 and 5, mice were treated by intraperitoneal injection with vehicle, or 10 mg/kg control Ab (IgG) or 10 mg/kg F^P+P^-L6^1+3^ (FLAg). The treatments were blinded to the investigators until the end of the experiment. Starting on day 2, vehicle or 50 mg/kg C59 was administered by gavage to the vehicle group or the two experimental groups (IgG and FLAg), respectively, twice a day with 8 hr interval for 4 days. On day 6, the mice were sacrificed. The whole intestinal tissue was harvested, cleaned with cold PBS, dehydrated with PBS, 30% sucrose, fixed with 4% paraformaldehyde and embedded in OCT. 8 μm OCT frozen sections were used for immunohistology. The intestinal EGFP crypts were analyzed using confocal microscopy (Zeiss LSM700).

## Data Availability

All data generated or analysed during this study are included in the manuscript and supporting files. FASTA sequence files have been provided for the FLAgs modalities we are describing in this study.

## Additional files

Supplementary file 1. FLAg amino acid sequences.

Supplementary file 2. Key Resources Table.

Supplementary file 3. FLAg nucleotide sequences.

Transparent reporting form
