# Supplementary material for: Tailored tetravalent antibodies potently and specifically activate Wnt/Frizzled pathways in cells, organoids and mice
Source: eLife. 2019 Aug 27;8:e46134. doi: 10.7554/eLife.46134 (PMC6711705; doi:10.7554/eLife.46134)
Supplement: Supplementary file 1. [file elife-46134-supp1.docx]

>FLAg 2+2_A

EVQLVESGGGLVQPGGSLRLSCAASGFNIYYSSIHWVRQAPGKGLEWVASIYPYYGYTYYADSVKGRFTISADTSKNTAYLQMNSLRAEDTAVYYCARYYHYGLDYWGQGTLVTVSSGGGGSDIQMTQSPSSLSASVGDRVTITCRASQSVSSAVAWYQQKPGKAPKLLIYSASSLYSGVPSRFSGSRSGTDFTLTISSLQPEDFATYYCQQSYWHSYLITFGQGTKVEIKLEDKTHTKVEPKTSDKTHTCPPCPAPELLGGPSVFLFPPKPKDTLMISRTPEVTCVVVDVSHEDPEVKFNWYVDGVEVHNAKTKPREEQYNSTYRVVSVLTVLHQDWLNGKEYKCKVSNKALPAPIEKTISKAKGQPREPMVFDLPPSREEMTKNQVSLWCMVKGFYPSDIAVEWESNGQPENNYKTTPPVLDSDGSFFLYSKLTVDKSRWQQGNVFSCSVMHEALHNHYTQKSLSLSPGKSGSETPGTSESATPESGGGEVQLVESGGGLVQPGGSLRLSCAASGFNISYSSIHWVRQAPGKGLEWVAYISSYYGYTYYADSVKGRFTISADTSKNTAYLQMNSLRAEDTAVYYCARAHYFPWAGAMDYWGQGTLVTVSSGGGGSDIQMTQSPSSLSASVGDRVTITCRASQSVSSAVAWYQQKPGKAPKLLIYSASSLYSGVPSRFSGSRSGTDFTLTISSLQPEDFATYYCQQYYWPITFGQGTKVEIK*

>FLAg 2-2_B

EVQLVESGGGLVQPGGSLRLSCAASGFNIYYSSIHWVRQAPGKGLEWVASIYPYYGYTYYADSVKGRFTISADTSKNTAYLQMNSLRAEDTAVYYCARYYHYGLDYWGQGTLVTVSSGGGGSDIQMTQSPSSLSASVGDRVTITCRASQSVSSAVAWYQQKPGKAPKLLIYSASSLYSGVPSRFSGSRSGTDFTLTISSLQPEDFATYYCQQSYWHSYLITFGQGTKVEIKLEDKTHTKVEPKSSDKTHNCPPCPAPELLGGPSVFLFPPKPKDTLMISRTPEVTCVVVDVSHEDPEVKFNWYVDGVEVHNAKTKPREEQYNSTYRVVSVLTVLHQDWLNGKEYKCKVSNKALPAPIEKTISKAKGQPREPQVYTLPPIRELMTSNQVSLSCAVKGFYPSDIAVEWESNGQPENNYKTTPPVLDSDGSFFLVSKLTVDKSRWQQGNVFSCSVMHEALHNHYTQKSLSLSPGKSGSETPGTSESATPESGGGEVQLVESGGGLVQPGGSLRLSCAASGFNISSYYIHWVRQAPGKGLEWVASIYSSYGYTSYADSVKGRFTISADTSKNTAYLQMNSLRAEDTAVYYCARTVRGSKKPYFSGWAMDYWGQGTLVTVSSGGGGSDIQMTQSPSSLSASVGDRVTITCRASQSVSSAVAWYQQKPGKAPKLLIYSASSLYSGVPSRFSGSRSGTDFTLTISSLQPEDFATYYCQQYSWGPFTFGQGTKVEIK*

>FLAg 7+7_A

EVQLVESGGGLVQPGGSLRLSCAASGFNISSSSMHWVRQAPGKGLEWVASIYSYYGSTYYADSVKGRFTISADTSKNTAYLQMNSLRAEDTAVYYCARWYGMDYWGQGTLVTVSSGGGGSDIQMTQSPSSLSASVGDRVTITCRASQSVSSAVAWYQQKPGKAPKLLIYSASSLYSGVPSRFSGSRSGTDFTLTISSLQPEDFATYYCQQPGSWYFPPITFGQGTKVEIKLEDKTHTKVEPKTSDKTHTCPPCPAPELLGGPSVFLFPPKPKDTLMISRTPEVTCVVVDVSHEDPEVKFNWYVDGVEVHNAKTKPREEQYNSTYRVVSVLTVLHQDWLNGKEYKCKVSNKALPAPIEKTISKAKGQPREPMVFDLPPSREEMTKNQVSLWCMVKGFYPSDIAVEWESNGQPENNYKTTPPVLDSDGSFFLYSKLTVDKSRWQQGNVFSCSVMHEALHNHYTQKSLSLSPGKSGSETPGTSESATPESGGGEVQLVESGGGLVQPGGSLRLSCAASGFNISYSSIHWVRQAPGKGLEWVAYISSYYGYTYYADSVKGRFTISADTSKNTAYLQMNSLRAEDTAVYYCARAHYFPWAGAMDYWGQGTLVTVSSGGGGSDIQMTQSPSSLSASVGDRVTITCRASQSVSSAVAWYQQKPGKAPKLLIYSASSLYSGVPSRFSGSRSGTDFTLTISSLQPEDFATYYCQQYYWPITFGQGTKVEIK*

>FLAg 7+7_B EVQLVESGGGLVQPGGSLRLSCAASGFNISSSSMHWVRQAPGKGLEWVASIYSYYGSTYYADSVKGRFTISADTSKNTAYLQMNSLRAEDTAVYYCARWYGMDYWGQGTLVTVSSGGGGSDIQMTQSPSSLSASVGDRVTITCRASQSVSSAVAWYQQKPGKAPKLLIYSASSLYSGVPSRFSGSRSGTDFTLTISSLQPEDFATYYCQQPGSWYFPPITFGQGTKVEIKLEDKTHTKVEPKSSDKTHNCPPCPAPELLGGPSVFLFPPKPKDTLMISRTPEVTCVVVDVSHEDPEVKFNWYVDGVEVHNAKTKPREEQYNSTYRVVSVLTVLHQDWLNGKEYKCKVSNKALPAPIEKTISKAKGQPREPQVYTLPPIRELMTSNQVSLSCAVKGFYPSDIAVEWESNGQPENNYKTTPPVLDSDGSFFLVSKLTVDKSRWQQGNVFSCSVMHEALHNHYTQKSLSLSPGKSGSETPGTSESATPESGGGEVQLVESGGGLVQPGGSLRLSCAASGFNISSYYIHWVRQAPGKGLEWVASIYSSYGYTSYADSVKGRFTISADTSKNTAYLQMNSLRAEDTAVYYCARTVRGSKKPYFSGWAMDYWGQGTLVTVSSGGGGSDIQMTQSPSSLSASVGDRVTITCRASQSVSSAVAWYQQKPGKAPKLLIYSASSLYSGVPSRFSGSRSGTDFTLTISSLQPEDFATYYCQQYSWGPFTFGQGTKVEIK*

>FLAg P+P_A EVQLVESGGGLVQPGGSLRLSCAASGFNIGSSSIHWVRQAPGKGLEWVASIYSAFASTSYADSVKGRFTISADTSKNTAYLQMNSLRAEDTAVYYCARYHFPFGFALDYWGQGTLVTVSSGGGGSDIQMTQSPSSLSASVGDRVTITCRASQSVSSAVAWYQQKPGKAPKLLIYSASSLYSGVPSRFSGSRSGTDFTLTISSLQPEDFATYYCQQGVYLFTFGQGTKVEIKLEDKTHTKVEPKTSDKTHTCPPCPAPELLGGPSVFLFPPKPKDTLMISRTPEVTCVVVDVSHEDPEVKFNWYVDGVEVHNAKTKPREEQYNSTYRVVSVLTVLHQDWLNGKEYKCKVSNKALPAPIEKTISKAKGQPREPMVFDLPPSREEMTKNQVSLWCMVKGFYPSDIAVEWESNGQPENNYKTTPPVLDSDGSFFLYSKLTVDKSRWQQGNVFSCSVMHEALHNHYTQKSLSLSPGKSGSETPGTSESATPESGGGEVQLVESGGGLVQPGGSLRLSCAASGFNISYSSIHWVRQAPGKGLEWVAYISSYYGYTYYADSVKGRFTISADTSKNTAYLQMNSLRAEDTAVYYCARAHYFPWAGAMDYWGQGTLVTVSSGGGGSDIQMTQSPSSLSASVGDRVTITCRASQSVSSAVAWYQQKPGKAPKLLIYSASSLYSGVPSRFSGSRSGTDFTLTISSLQPEDFATYYCQQYYWPITFGQGTKVEIK*

>FLAg P+P_B EVQLVESGGGLVQPGGSLRLSCAASGFNIGSSSIHWVRQAPGKGLEWVASIYSAFASTSYADSVKGRFTISADTSKNTAYLQMNSLRAEDTAVYYCARYHFPFGFALDYWGQGTLVTVSSGGGGSDIQMTQSPSSLSASVGDRVTITCRASQSVSSAVAWYQQKPGKAPKLLIYSASSLYSGVPSRFSGSRSGTDFTLTISSLQPEDFATYYCQQGVYLFTFGQGTKVEIKLEDKTHTKVEPKSSDKTHNCPPCPAPELLGGPSVFLFPPKPKDTLMISRTPEVTCVVVDVSHEDPEVKFNWYVDGVEVHNAKTKPREEQYNSTYRVVSVLTVLHQDWLNGKEYKCKVSNKALPAPIEKTISKAKGQPREPQVYTLPPIRELMTSNQVSLSCAVKGFYPSDIAVEWESNGQPENNYKTTPPVLDSDGSFFLVSKLTVDKSRWQQGNVFSCSVMHEALHNHYTQKSLSLSPGKSGSETPGTSESATPESGGGEVQLVESGGGLVQPGGSLRLSCAASGFNISSYYIHWVRQAPGKGLEWVASIYSSYGYTSYADSVKGRFTISADTSKNTAYLQMNSLRAEDTAVYYCARTVRGSKKPYFSGWAMDYWGQGTLVTVSSGGGGSDIQMTQSPSSLSASVGDRVTITCRASQSVSSAVAWYQQKPGKAPKLLIYSASSLYSGVPSRFSGSRSGTDFTLTISSLQPEDFATYYCQQYSWGPFTFGQGTKVEIK*

>FLAg 5+5_A EVQLVESGGGLVQPGGSLRLSCAASGFNISYSSIHWVRQAPGKGLEWVASIYPSYSSTYYADSVKGRFTISADTSKNTAYLQMNSLRAEDTAVYYCARYYAMDYWGQGTLVTVSSGGGGSDIQMTQSPSSLSASVGDRVTITCRASQSVSSAVAWYQQKPGKAPKLLIYSASSLYSGVPSRFSGSRSGTDFTLTISSLQPEDFATYYCQQAFYYPITFGQGTKVEIKLEDKTHTKVEPKTSDKTHTCPPCPAPELLGGPSVFLFPPKPKDTLMISRTPEVTCVVVDVSHEDPEVKFNWYVDGVEVHNAKTKPREEQYNSTYRVVSVLTVLHQDWLNGKEYKCKVSNKALPAPIEKTISKAKGQPREPMVFDLPPSREEMTKNQVSLWCMVKGFYPSDIAVEWESNGQPENNYKTTPPVLDSDGSFFLYSKLTVDKSRWQQGNVFSCSVMHEALHNHYTQKSLSLSPGKSGSETPGTSESATPESGGGEVQLVESGGGLVQPGGSLRLSCAASGFNISYSSIHWVRQAPGKGLEWVAYISSYYGYTYYADSVKGRFTISADTSKNTAYLQMNSLRAEDTAVYYCARAHYFPWAGAMDYWGQGTLVTVSSGGGGSDIQMTQSPSSLSASVGDRVTITCRASQSVSSAVAWYQQKPGKAPKLLIYSASSLYSGVPSRFSGSRSGTDFTLTISSLQPEDFATYYCQQYYWPITFGQGTKVEIK*

>FLAg 5+5_B EVQLVESGGGLVQPGGSLRLSCAASGFNISYSSIHWVRQAPGKGLEWVASIYPSYSSTYYADSVKGRFTISADTSKNTAYLQMNSLRAEDTAVYYCARYYAMDYWGQGTLVTVSSGGGGSDIQMTQSPSSLSASVGDRVTITCRASQSVSSAVAWYQQKPGKAPKLLIYSASSLYSGVPSRFSGSRSGTDFTLTISSLQPEDFATYYCQQAFYYPITFGQGTKVEIKLEDKTHTKVEPKSSDKTHNCPPCPAPELLGGPSVFLFPPKPKDTLMISRTPEVTCVVVDVSHEDPEVKFNWYVDGVEVHNAKTKPREEQYNSTYRVVSVLTVLHQDWLNGKEYKCKVSNKALPAPIEKTISKAKGQPREPQVYTLPPIRELMTSNQVSLSCAVKGFYPSDIAVEWESNGQPENNYKTTPPVLDSDGSFFLVSKLTVDKSRWQQGNVFSCSVMHEALHNHYTQKSLSLSPGKSGSETPGTSESATPESGGGEVQLVESGGGLVQPGGSLRLSCAASGFNISSYYIHWVRQAPGKGLEWVASIYSSYGYTSYADSVKGRFTISADTSKNTAYLQMNSLRAEDTAVYYCARTVRGSKKPYFSGWAMDYWGQGTLVTVSSGGGGSDIQMTQSPSSLSASVGDRVTITCRASQSVSSAVAWYQQKPGKAPKLLIYSASSLYSGVPSRFSGSRSGTDFTLTISSLQPEDFATYYCQQYSWGPFTFGQGTKVEIK*

>FLAg 4+4_A EVQLVESGGGLVQPGGSLRLSCAASGFNSSFYFMHWVRQAPGKGLEWVATVYPYLDYTYYADSVKGRFTISADTSKNTAYLQMNSLRAEDTAVYYCARAFPGSYHPMDYWGQGTLVTVSSGGGGSDIQMTQSPSSLSASVGDRVTITCRASQSVSSAVAWYQQKPGKAPKLLIYSASSLYSGVPSRFSGSRSGTDFTLTISSLQPEDFATYYCQQSSYSLITFGQGTKVEIKLEDKTHTKVEPKTSDKTHTCPPCPAPELLGGPSVFLFPPKPKDTLMISRTPEVTCVVVDVSHEDPEVKFNWYVDGVEVHNAKTKPREEQYNSTYRVVSVLTVLHQDWLNGKEYKCKVSNKALPAPIEKTISKAKGQPREPMVFDLPPSREEMTKNQVSLWCMVKGFYPSDIAVEWESNGQPENNYKTTPPVLDSDGSFFLYSKLTVDKSRWQQGNVFSCSVMHEALHNHYTQKSLSLSPGKSGSETPGTSESATPESGGGEVQLVESGGGLVQPGGSLRLSCAASGFNISYSSIHWVRQAPGKGLEWVAYISSYYGYTYYADSVKGRFTISADTSKNTAYLQMNSLRAEDTAVYYCARAHYFPWAGAMDYWGQGTLVTVSSGGGGSDIQMTQSPSSLSASVGDRVTITCRASQSVSSAVAWYQQKPGKAPKLLIYSASSLYSGVPSRFSGSRSGTDFTLTISSLQPEDFATYYCQQYYWPITFGQGTKVEIK*

>FLAg 4+4_B EVQLVESGGGLVQPGGSLRLSCAASGFNSSFYFMHWVRQAPGKGLEWVATVYPYLDYTYYADSVKGRFTISADTSKNTAYLQMNSLRAEDTAVYYCARAFPGSYHPMDYWGQGTLVTVSSGGGGSDIQMTQSPSSLSASVGDRVTITCRASQSVSSAVAWYQQKPGKAPKLLIYSASSLYSGVPSRFSGSRSGTDFTLTISSLQPEDFATYYCQQSSYSLITFGQGTKVEIKLEDKTHTKVEPKSSDKTHNCPPCPAPELLGGPSVFLFPPKPKDTLMISRTPEVTCVVVDVSHEDPEVKFNWYVDGVEVHNAKTKPREEQYNSTYRVVSVLTVLHQDWLNGKEYKCKVSNKALPAPIEKTISKAKGQPREPQVYTLPPIRELMTSNQVSLSCAVKGFYPSDIAVEWESNGQPENNYKTTPPVLDSDGSFFLVSKLTVDKSRWQQGNVFSCSVMHEALHNHYTQKSLSLSPGKSGSETPGTSESATPESGGGEVQLVESGGGLVQPGGSLRLSCAASGFNISSYYIHWVRQAPGKGLEWVASIYSSYGYTSYADSVKGRFTISADTSKNTAYLQMNSLRAEDTAVYYCARTVRGSKKPYFSGWAMDYWGQGTLVTVSSGGGGSDIQMTQSPSSLSASVGDRVTITCRASQSVSSAVAWYQQKPGKAPKLLIYSASSLYSGVPSRFSGSRSGTDFTLTISSLQPEDFATYYCQQYSWGPFTFGQGTKVEIK*
