# Supplementary material for: Tailored tetravalent antibodies potently and specifically activate Wnt/Frizzled pathways in cells, organoids and mice
Source: eLife. 2019 Aug 27;8:e46134. doi: 10.7554/eLife.46134 (PMC6711705; doi:10.7554/eLife.46134)
Supplement: Supplementary file 2. [file elife-46134-supp2.docx]

| **Key Resources Table** | | | | |
| --- | --- | --- | --- | --- |
| **Reagent type (species) or resource** | **Designation** | **Source or reference** | **Identifiers** | **Additional information** |
| Lgr5-EGFP-IRES-creERT2, B6 (mouse) | B6.129P2-Lgr5^tm1(cre/ERT2)Cle^/J | The Jackson Laboratory | 008875 |  |
| B6, B6 (mouse) | C57BL/6J | The Jackson Laboratory | 000664 |  |
| cell line (HPAF-II) | human pancreatic adenocarcinoma | ATCC | CRL-1997 | PDAC model |
| cell line (HEK293T) | Human embryonic kidney cells | ATCC | CRL-3216 | Transfected with hFZD receptors |
| cell line  (CHO-K1) | Chinese hamster ovary cells | ATCC | CCL-61 | Transfected with GPI-linked FZD CRD (Steinhart Z. et al. 2017) |
| cell line (human) | H1 human embryonic stem cells |  |  | Sex: male |
| biological sample (Wnt3 CM) | WNT3A CM from L cells |  |  | WNT3A conditioned media |
| peptide, recombinant protein | FZD1 | R&D Systems | 5988-FZ-050 | Fc hIgG1 fusion |
| peptide, recombinant protein | FZD2 | R&D Systems | 1307-FZ-050 | Fc hIgG1 fusion |
| peptide, recombinant protein | FZD4 | R&D Systems | 5847-FZ-050 | Fc hIgG1 fusion |
| peptide, recombinant protein | FZD5 | R&D Systems | 1617-FZ-050 | Fc hIgG1 fusion |
| peptide, recombinant protein | FZD6 | In house | 19-132, Uniprot O60353-1 | Fc hIgG1 fusion, pFUSE (Invivogen) |
| peptide, recombinant protein | FZD7 | R&D Systems | 6178-FZ-050 | Fc-fusion |
| peptide, recombinant protein | FZD8 | R&D Systems | 6129-FZ-050 | Fc hIgG1 fusion |
| peptide, recombinant protein | FZD9 | R&D Systems | 9175-FZ-050 | Fc hIgG1 fusion |
| peptide, recombinant protein | FZD10 | R&D Systems | 3459-FZ-050 | Fc hIgG1 fusion |
| peptide, recombinant protein | LRP6 | R&D Systems | 1505-LR-025 |  |
| peptide, recombinant protein | LRP5, mouse | R&D Systems | 29-LR-025 |  |
| Chemical compound, drug | LGK974 | Cayman Chemicals | 14072 |  |
| Chemical compound, drug | C59 | Dalriada Therapeutics | custom synthesis |  |
| antibody | Brachyury (goat polyclonal) | R&D systems | AF2085 | IF dilution (1:100) |
| antibody | Oct3-4 (mouse monoclonal) | Santa Cruz | SC5279 | IF dilution (1:100)  WB dilution (1:1000) |
| antibody | β-tubulin (mouse monoclonal) | Hybridoma bank | E7 | WB dilution (1:1000) |
| chemical compound, drug | CHIR99021 | Sigma-Aldrich | SML1046-5MG |  |
| other | DAPI | Sigma-Aldrich | **D9542** |  |
| organoids  (small intestine) | Small intestine (C57BL/6J mouse) | In house |  | Isolation protocol: [(O’Rourke et al. 2016)](https://paperpile.com/c/bqLcGj/67pN) |
| organoids  (human colon) | Human colon organoids | Clevers lab |  |  |
| recombinant protein | Epidermal growth factor (mouse) | Thermofisher | PMG8043 | organoid culture |
| recombinant protein | Noggin (mouse) | Peprotech | 250-38 | organoid culture |
| compound, drug | y-27632 (ROCK inhibitor) | Sigma | Y0503 | organoid culture |
| recombinant protein | Gastrin | Sigma | G9145 | organoid culture |
| compound, drug | A8301 | Tocris Bioscience | 2939 | organoid culture |
| compound, drug | SB202190 | Sigma | S70767 | organoid culture |
| Commercial assay, kit | Cell titer Glo 3D | Promega | G9681 | organoid viability |
| compound, drug | B27 | Thermofisher | 17504044 | organoid culture |
| compound, drug | N2 | Thermofisher | 17502001 | organoid culture |
