# Supplementary material for: Tailored tetravalent antibodies potently and specifically activate Wnt/Frizzled pathways in cells, organoids and mice
Source: eLife. 2019 Aug 27;8:e46134. doi: 10.7554/eLife.46134 (PMC6711705; doi:10.7554/eLife.46134)
Supplement: Peer review history

# Decision letter

**DOI:** 10.7554/eLife.46134.027

**Affiliations:** California Institute of TechnologyUnited States

In the interests of transparency, eLife includes the editorial decision letter and accompanying author responses. A lightly edited version of the letter sent to the authors after peer review is shown, indicating the most substantive concerns; minor comments are not usually included.

[Editors’ note: this article was originally rejected after discussions between the reviewers, but the authors were invited to resubmit after an appeal against the decision.]

Thank you for choosing to send your work, "Tailored tetravalent antibodies for potent and specific activation of Wnt/Frizzled pathways in vivo", for consideration at *eLife*. Although the work is of interest, we regret to inform you that the findings at this stage are too preliminary for further consideration at *eLife*.

Specifically, we appreciate the importance of this new set of tools that could enable ground-breaking studies by many groups to better understand the specificity and combinatorial complexity of Wnt:Receptor binding and signaling (at least the first, early steps). On the other hand, it was felt that creating a fusion protein to aggregate two signaling entities is not a new strategy. The in vivo experiments seem quite limited and need further quantification and statistics. The paper could be expanded to show how their "FLAg" reagent provides higher resolution insight into activity and/or delineates a specific activity via a specific FZD. Given these concerns, on balance, we feel that you should come back with a new paper with further controls and more in vivo and in vitro experiments that are well quantified. This new submission could then be considered as a Tools and Resource and every effort would be made to return the paper to the same reviewers. The full reviews are listed below for your information.

*Reviewer #1:*

Tao et al. present a new type of Wnt mimic called FLAg (for Frizzled Lrp Agonist). FLAgs are tetravalent antibodies engineered to simultaneously engage a Frizzled receptor and LRP5/6 co-receptor in ways that aggregate them to activate downstream Wnt signals. The specificities of the Flags were developed through phage display screening and this study shows that specific FZD receptors can be engaged, allowing one to interrogate downstream events specific to one type of Wnt and one type of FZD receptor. The authors show activity of these reagents through in vitro reporter assays (via β-catenin levels and/or phosphorylated dishevelled), small intestinal organoid cultures, and in an in vivo reporter mouse where intestinal stem cells are labeled by LGR5-GFP. While the latter two assays do not necessarily report on receptor specificity, they do demonstrate viable FLAg activity in a more complex environment. Given that it is difficult to purify active Wnt ligands, FLAgs represent new tools that can interrogate the Wnt:FZD space much better than any reagent developed so far. The manuscript is mainly focused on development and validation of these intriguing reagents, with some mechanistic insight (albeit minor). Overall this study is an important advance that will be of interest to i) research groups using organoids to study normal and diseased tissue, ii) developmental biologists that find specific Wnts or Frizzleds important in their system and iii) protein engineers in the therapeutics community – a community interested in developing reagents that either inhibit or activate Wnt signaling.

*Reviewer #2:*

In this study, the authors reported the newly synthesized tetravalent antibodies 'FLAgs', which are water soluble, modular and engineerable FZD/LRP agonists, and could activate Wnt-β-catenin signaling via formation of a complex with specific FZD and LRP5/6 to control the differentiation of PSCs, sustain mouse small intestinal organoids in vitro, and maintain the mouse small intestinal stem cell functions in vivo. Although the present results contain interest findings, but as the authors cited (Nature, 2017 May 11;545(7653):234-237), it has been already reported that the bivalent FZD/LRP agonists, which are also water soluble, easily produced, modular and engineerable, bind simultaneously various FZD and LRP6, and activate the Wnt-β-catenin pathway to direct the differentiation of MSCs, support the growth of various human tissue organoids in vitro, and regulate the metabolic zonation of liver in mouse model. It seems to this reviewer that the present study lacks novelty.

The authors proposed some advances of their FLAgs compared with the bivalent FZD/LRP agonists. For example, due to their tetravalent structure, FLAgs could recognize the more diverse targets in specific signaling contexts by the replacement of one FZD binding site with a module targeting another paratope. However, as shown in Figure 2D, FP+0 exhibited only about 1/10 Luciferase activity when compared with FP+P. Therefore, the authors' proposal seems not to be promising. In addition, the authors also proposed that since FLAgs contain the IgG-Fc component in their structure, they would show the antibody-like characters in vivo, that is, good bioavailability, long half-life time, and low immunogenicity. However, the presented data were only in-vitro binding assay. It is hard to emphasize the Ab-like character of FLAgs as an advantage.

Other detailed comments are as follows.

1) Figures 1E and F and 2D: The authors should add the positive- and negative-control experiments. Treatment with purified Wnt3a proteins as positive control and that with purified DKK1 proteins as negative control assay would be appropriate.

2) Figure 3A and B: The actual measured values of Kd of BLI assay should be presented to directly compare to the binding affinity of each Fx to FZD paratopes. There is possibility that F2 shows little binding affinity to FZD2 when compared with F5 to FZD5 in Figure 3A and that F2+5 shows little binding affinity to FZD5 when compared with F5+5 to FZD5 in Figure 3B.

3) Figure 3C: The authors should use other cell lines, such as L cells (expressing FZD7), A375 and SH-SY5Y cells (expressing Fzd2), and A549 cells (expressing Fzd2, 4, 6, and 8) to validate FLAgs other than F5+5 or FP+P.

4) Figure 4C: Results are not convincing. Quantification and statistical analyses are absolutely required.

*Reviewer #3:*

Sidhu and colleagues describe a platform to create selective FZD agonists by combining different FZD and LRP6 binders selected by phage display into tetravalent complexes that potently activate Wnt signaling pathways. They characterized >160 different FZD binders for their binding affinities for all ten FZD receptors. They further combined multiple FZD binders, together with binders of LRP5/6 into tetravalent complexes ("FLAgs") that showed similar affinity and activity as 'native' Wnt proteins. By combining different FZD binders, FLAgs can activate different FZD/co-receptor complexed and have the potential to selectively mimic the activity of different Wnt proteins. They further demonstrate the potency of FLAgs ex vivo and in vivo.

This is an important study that describes a platform for rational design of Wnt signaling agonists with broad applicability for mechanistic studies, as well as for regenerative medicine. While 'artificial' Wnt agonists have been described before, the contribution of this study is the rational design of Wnt agonists, with the potential for further extension due to the tetravalent design of the FLAgs.

I have a few comments that I feel the authors should address prior to publication:

1) FLAgs show a bell-shaped activity curve (Figure 1D), similar to previous Wnt agonists. It would be helpful for the reader to know whether a similar activity curve can be observed in other assays, e.g. for organoid growth

2) Figure 3C consists of low quality images, these should be replaced with better images.

3) I might have missed it, but I could not find the epitope sequences of Fabs used in this study. Sequences for experiments shown in Figure 1A and others should be provided in the supplement or submitted to a public database to ensure the ability of others to reproduce the experiments. Optimally, the supplement should also contain the full sequence of the tetravalent binders constructed in this study.

[Editors’ note: what now follows is the decision letter after the authors submitted for further consideration.]

Thank you for submitting your article "Tailored tetravalent antibodies potently and specifically activate Wnt/Frizzled pathways in cells, organoids and mice." for consideration by *eLife*. Your article has been reviewed by two peer reviewers, and the evaluation has been overseen by Marianne Bronner as the Senior/Reviewing Editor. The reviewers have opted to remain anonymous.

The reviewers have discussed the reviews with one another and the Reviewing Editor has drafted this decision to help you prepare a revised submission. As you will see from the reviews below, reviewer 3 is not satisfied with the methods and requests (again) full disclosure of FLAg sequences and constructs. This seems particularly important for a resource paper and I support the reviewer's request as mandatory for publication. Please see the full reviews below for further details.

*Reviewer #1:*

This revision addresses the major concerns of this reviewer.

*Reviewer #3:*

The revised manuscript addressed several comments in my previous review, however, a comment that I consider essential for a technology / resource-focused publication has not been addressed.

Without full disclosure of FLAg sequences and constructs to enable independent validation, the manuscript in my view should not be published. The current disclosure of methods and sequences does not live up to the standards that *eLife* should promote.

In my past review, I commented:

"3) I might have missed it, but I could not find the epitope sequences of Fabs used in this study. Sequences for experiments shown in Figure 1A and others should be provided in the supplement or submitted to a public database to ensure the ability of others to reproduce the experiments. Optimally, the supplement should also contain the full sequence of the tetravalent binders constructed in this study."

The authors responded:

"We have filed a patent describing the molecules that contains the sequence information for some of the FLAg. We recently pursued a PCT application and the patent should be published very soon. We are now including the sequences for the FLAg in this manuscript for which we show functional activity, so that the community could make these molecules. We are also working with a reagents company to provide proteins to the community"

I find their answer to be evasive. A scientific publication as a permanent record should fully disclose all information that is required to reproduce the findings described in the manuscript.

In the supplementary data, I only found amino acid sequences of selected epitopes but full nucleotide sequence data of constructs as used e.g. Figure 1A (and which was requested), is currently missing.

The authors should extend the Materials and methods section, (a) fully explain how the FLAgs are constructed and (b) provide full nucleotide sequences of vectors and FLAgs to enable reproducibility. The authors should also provide a supplementary table indicating which nucleotide sequence derived product has been used in which figure.

It is not acceptable to reference an on-going patent application or negotiations with a reagent company in lieu of fully documented reagents.

The authors also declared that they do not have a conflict of interest. In the light of their answers to the reviewer's question, the authors might want to reconsider whether this statement is complete.

# Reply: Author response

**DOI:** 10.7554/eLife.46134.028

[Editors’ note: the author responses to the first round of peer review follow.]

We are excited from the assessment of your editorial team that the tools reported in our study will enable groundbreaking studies. We are also excited with the overall positive reviews of all three reviewers and their recognition that this will change our ability to study Wnt signalling. Admittedly, as you pointed out, bivalent molecules and soluble “surrogate” Wnt ligands have been reported by the Garcia group. However, there is several very important distinctions between the “Garcia” molecule and the FLAgs reported in our manuscript. First, the FLAgs are all antibody based and as such are long-lived in vivo and as we demonstrate can be injected as proteins and maintain activity to activate endogenous signaling in the mouse. In order to perform in vivo studies, Garcia et al. had to deliver the Wnt surrogate using adenoviral vectors, presumably because of the instability and the lack of bioactivity of their molecule (PMID: 28467818). Second, we show clearly that tetravalent engagement of Wnt/Fzd is required for optimal activity and to accurately mimic the natural signalling complex. The Garcia molecules are bivalent, and thus, the tetravalent FLAgs provide higher activity. Third, the tetravalent and modular nature of the FLAgs provides a facile means to build virtually any FZD monospecific or bispecific agonist by simple engineering, and thus, the FLAg approach opens up vast possibilities for mimicking natural Wnt ligands and extending to specificities beyond what is achieved by nature. We believe that in addition to providing powerful new research tools, FLAg will form first-in-class novel medicine that will promote Wnt signaling for regenerative medicine applications.

We are thankful to Reviewer #1, who recognizes that our study is an important and exciting advance that will impact not only researchers in the Wnt community but also more broadly the expanding number of researchers interested/using tissue organoids and the protein engineering community.

Reviewer #2:

*In this study, the authors reported the newly synthesized tetravalent antibodies 'FLAgs', which are water soluble, modular and engineerable FZD/LRP agonists, and could activate Wnt-β-catenin signaling via formation of a complex with specific FZD and LRP5/6 to control the differentiation of PSCs, sustain mouse small intestinal organoids* in vitro*, and maintain the mouse small intestinal stem cell functions* in vivo*. Although the present results contain interest findings, but as the authors cited (Nature, 2017 May 11;545(7653):234-237), it has been already reported that the bivalent FZD/LRP agonists, which are also water soluble, easily produced, modular and engineerable, bind simultaneously various FZD and LRP6, and activate the Wnt-β-catenin pathway to direct the differentiation of MSCs, support the growth of various human tissue organoids* in vitro, and regulate the metabolic zonation of liver in mouse model. It seems to this reviewer that the present study lacks novelty.

*The authors proposed some advances of their FLAgs compared with the bivalent FZD/LRP agonists. For example, due to their tetravalent structure, FLAgs could recognize the more diverse targets in specific signaling contexts by the replacement of one FZD binding site with a module targeting another paratope. However, as shown in Figure 2D, FP+0 exhibited only about 1/10 Luciferase activity when compared with FP+P. Therefore, the authors' proposal seems not to be promising. In addition, the authors also proposed that since FLAgs contain the IgG-Fc component in their structure, they would show the antibody-like characters* in vivo*, that is, good bioavailability, long half-life time, and low immunogenicity. However, the presented data were only in-vitro binding assay. It is hard to emphasize the Ab-like character of FLAgs as an advantage.*

We appreciate that reviewer #2 thinks our study contains interesting findings. We understand that the tetravalent FLAg are similar in principle to the reported “surrogate” Wnt molecule described by Garcia, however we believe that the reviewer missed the most important difference in that the FLAg are active “in vivo” when injected as proteins whereas the Garcia surrogate needs to be delivered with adenovirus. We believe this bioactivity is provided by the antibody design of the FLAg, which confers stability and long half lives in vivo.

With respect to the advantage of the tetravalent modality of the FLAg, we foresee that the principal advantage will be the modularity that this is providing. Indeed, any of the Frizzled antibody can easily be subcloned into the two Frizzled binding sites thereby providing the ability to tailor the desired specificity using the available Frizzled antibodies. For example, combining selective FZD2 and FZD5 antibodies in one FLAg would lead to a bispecific FLAg specifically activating both of these FZD receptors. In addition, since the F^P+0^-L6^1+3^ molecule still exhibited 35-40 folds activation (admittedly 1/10 of the parent molecule) we suggested that using this binding site to target the molecule to a specific cellular context or tissue using another antibody could provide more specificity and limit toxicity when delivered in vivo. Off course this remains to be seen but it demonstrates the modularity and versatility of the tetravalent FLAg.

Another aspect that we did not discuss in the manuscript is that by disabling one of the FZD targeting paratopes in the F^P+0^-L6^1+3^ molecule, we in essence generates a bivalent molecule similar to the Wnt surrogate reported by Garcia. As pointed out by the reviewer, this molecule only exhibit 10% of the maximal activity obtained with the tetravalent FLAg, suggesting that engagement of a higher order FZD:LRP5/6 oligomers may provide higher signalling activity. Interestingly, a recent study (PMID: 31036956) suggests that Wnt proteins may be favoring the formation of a Frizzled and LRP5/6 oligomeric complex containing 2 Frizzled and 2 LRP5/6 proteins. The tetravalent FLAg molecule may therefore better mimic Wnt proteins and provide higher signaling activity.

Other detailed comments are as follows.

1) Figures 1E and F and 2D: The authors should add the positive- and negative-control experiments. Treatment with purified Wnt3a proteins as positive control and that with purified DKK1 proteins as negative control assay would be appropriate.

Maybe this was not clearly described and highlighted but we have performed a careful analysis comparing the activity of the pan-FLAg molecule to purified Wnt3A and to the GSK3 inhibitor CHIR99021 that we included in Figure 1—figure supplement 1I. Since the pathway is not active in the absence of ligand we don’t see how the addition of the inhibitor DKK1 would add anything.

2) Figure 3A and B: The actual measured values of Kd of BLI assay should be presented to directly compare to the binding affinity of each Fx to FZD paratopes. There is possibility that F2 shows little binding affinity to FZD2 when compared with F5 to FZD5 in Figure 3A and that F2+5 shows little binding affinity to FZD5 when compared with F5+5 to FZD5 in Figure 3B.

This is an interesting point raised by the reviewer. We now provide in Figure 3—figure supplement 1B and C the BLI data and the measured Kd for the FZD5 specific FLAg and all the bispecific FZD5 FLAg presented in Figure 3B.

3) Figure 3C: The authors should use other cell lines, such as L cells (expressing FZD7), A375 and SH-SY5Y cells (expressing Fzd2), and A549 cells (expressing Fzd2, 4, 6, and 8) to validate FLAgs other than F5+5 or FP+P.

This is a good point raised by reviewer #2. To extend our findings to other FLAgs we used HEK293 cells for which systematic CRISPR knockout of Frizzled receptors was conducted and revealed that FZD1, FZD2 and FZD7 were redundantly required for activation of βcatenin signalling in response to WNT3/WNT3A (PMID: 28733458). Supporting this data, we demonstrate that FZD2 and FZD7 but not FZD4 and FZD5 specific FLAg molecules activate β-catenin signalling in these cells (Figure 3D).

4) Figure 4C: Results are not convincing. Quantification and statistical analyses are absolutely required.

For Figure 4C, we provided a quantification of the cell viability in Figure 4D using CellTiter-glo. There is very little signal upon treatment of the organoids with LGK974 confirming previous results that the viability and growth of mouse small intestinal organoids depend on Wnt secreted by the paneth cells. Addition of WNT3A conditioned media or the pan-FLAg completely rescued the growth of organoids in the presence of LGK974. We feel that the celltiter-glo viability quantification is adequate and the results are black and white. Perhaps the reviewer was referring to the quality of the pictures, which admittedly are not by themselves convincing, but we feel that with the viability quantification this is very clear. We have added the statistics on the cell viability quantification and are now providing zoom-in inset pictures to showcase the viability.

Reviewer #3:

[…] I have a few comments that I feel the authors should address prior to publication:

1) FLAgs show a bell-shaped activity curve (Figure 1D), similar to previous Wnt agonists. It would be helpful for the reader to know whether a similar activity curve can be observed in other assays, e.g. for organoid growth

We performed a dose response curve for the FLAg for growth of mouse intestinal organoids and show no difference in growth rate between 0.1nM and 100nM suggesting that these doses are saturated and that even if there is a “bell shape” relationship in the dose response, the residual signaling activity at the highest dose of FLAg is likely more than enough to sustain growth (see Author response image 1). Of note, we only observe a 30-45% reduction of maximal signaling activity with large amount of FLAg (Figure 1D), so the remaining activity with high concentration of FLAg is likely more than enough to sustain organoid growth. Another consideration is the presence of Rspondin in the conditioned media that amplify Wnt-βcatenin signaling activity and suggesting that very little exogenous Wnt is needed. In fact, in the dose response for the pan-FLAg in the mouse intestinal organoids assay, as little as 100pM seems to be sufficient to maximally sustain the growth.

**Author response image 1.**

2) Figure 3C consists of low quality images, these should be replaced with better images.

Reviewer #3 probably referred to Figure 4C. This was also raised by reviewer #1. We now provide higher magnification figures in cropped inset images to make the point clearer.

3) I might have missed it, but I could not find the epitope sequences of Fabs used in this study. Sequences for experiments shown in Figure 1A and others should be provided in the supplement or submitted to a public database to ensure the ability of others to reproduce the experiments. Optimally, the supplement should also contain the full sequence of the tetravalent binders constructed in this study.

We have filed a patent describing the molecules that contains the sequence information for some of the FLAg. We recently pursued a PCT application and the patent should be published very soon. We are now including the sequences for the FLAg in this manuscript for which we show functional activity, so that the community could make these molecules. We are also working with a reagents company to provide proteins to the community.

[Editors’ note: the author responses to the re-review follow.]

The reviewers have discussed the reviews with one another and the Reviewing Editor has drafted this decision to help you prepare a revised submission. As you will see from the reviews below, reviewer 3 is not satisfied with the methods and requests (again) full disclosure of FLAg sequences and constructs. This seems particularly important for a resource paper and I support the reviewer's request as mandatory for publication. Please see the full reviews below for further details.

Reviewer #3:

The revised manuscript addressed several comments in my previous review, however, a comment that I consider essential for a technology / resource-focused publication has not been addressed.

Without full disclosure of FLAg sequences and constructs to enable independent validation, the manuscript in my view should not be published. The current disclosure of methods and sequences does not live up to the standards that eLife should promote.

In my past review, I commented:

"3) I might have missed it, but I could not find the epitope sequences of Fabs used in this study. Sequences for experiments shown in Figure 1A and others should be provided in the supplement or submitted to a public database to ensure the ability of others to reproduce the experiments. Optimally, the supplement should also contain the full sequence of the tetravalent binders constructed in this study."

The authors responded:

"We have filed a patent describing the molecules that contains the sequence information for some of the FLAg. We recently pursued a PCT application and the patent should be published very soon. We are now including the sequences for the FLAg in this manuscript for which we show functional activity, so that the community could make these molecules. We are also working with a reagents company to provide proteins to the community"

A scientific publication as a permanent record should fully disclose all information that is required to reproduce the findings described in the manuscript.

In the supplementary data, I only found amino acid sequences of selected epitopes but full nucleotide sequence data of constructs as used e.g. Figure 1A (and which was requested), is currently missing.

The authors should extend the Materials and methods section, (a) fully explain how the FLAgs are constructed and (b) provide full nucleotide sequences of vectors and FLAgs to enable reproducibility. The authors should also provide a supplementary table indicating which nucleotide sequence derived product has been used in which figure.

It is not acceptable to reference an on-going patent application or negotiations with a reagent company in lieu of fully documented reagents.

The authors also declared that they do not have a conflict of interest. In the light of their answers to the reviewer's question, the authors might want to reconsider whether this statement is complete.

Thank you for guiding us into modifying our manuscript to meet the standards of *eLife*. As stated previously, we are providing full sequence information for the molecules that are fully characterized and which exhibit functional activity.

At the request of one of your reviewers, we have now removed the data that described unvalidated preliminary antibody fragments as building blocks. As explain before, we are uncomfortable and it would be scientifically irresponsible to provide sequences for these reagents. We included them for illustrative purposes to explain the full potential of the platform. The removal of this data does not impact the conclusions reached in the manuscript.

The only changes to the figures are:

1) Figure 1A was replaced with a detailed biophysical characterization of the pan-Frizzled Fab for which we provide sequence.

2) Figure 3A, 3B and Figure 3—figure supplement 1 have been slightly modified to remove data from unvalidated building blocks for which we did not provide functional support.

As requested, we also have added as competing interest in the authors info that Tao, Angers and Sidhu have filed a provisional application related to the molecules described in the manuscript.
